# The OsmiR396–OsGRF8–OsF3H‐flavonoid pathway mediates resistance to the brown planthopper in rice (*Oryza sativa*)

**DOI:** 10.1111/pbi.13091

**Published:** 2019-03-13

**Authors:** Zhengyan Dai, Jiang Tan, Cong Zhou, Xiaofang Yang, Fang Yang, Shijuan Zhang, Shichen Sun, Xuexia Miao, Zhenying Shi

**Affiliations:** ^1^ Key Laboratory of Insect Developmental and Evolutionary Biology Institute of Plant Physiology and Ecology Shanghai Institutes for Biological Sciences Chinese Academy of Sciences Shanghai China; ^2^ State Key Laboratory of Hybrid Rice College of Life Sciences Wuhan University Wuhan China; ^3^ University of Chinese Academy of Sciences Shanghai China; ^4^ Shandong Province Key Laboratory of Life‐Organic Analysis Qufu Normal University Qufu China; ^5^ Institute of Crop Cultivation and Tillage Heilongjiang Academy of Agricultural Sciences & Northern Japonica Rice Molecular Breeding Joint Research Center Chinese Academy of Sciences Haerbin China

**Keywords:** OsmiR396, brown planthopper, flavonoid, rice (*Oryza sativa*), *OsF3H*

## Abstract

Multi‐functional microRNAs (miRNAs) are emerging as key modulators of plant–pathogen interactions. Although the involvement of some miRNAs in plant–insect interactions has been revealed, the underlying mechanisms are still elusive. The brown planthopper (BPH) is the most notorious rice (*Oryza sativa*)‐specific insect that causes severe yield losses each year and requires urgent biological control. To reveal the miRNAs involved in rice–BPH interactions, we performed miRNA sequencing and identified BPH‐responsive OsmiR396. Sequestering OsmiR396 by overexpressing target mimicry (MIM396) in three genetic backgrounds indicated that OsmiR396 negatively regulated BPH resistance. Overexpression of one BPH‐responsive target gene of OsmiR396, growth regulating factor 8 (*OsGRF8*), showed resistance to BPH. Furthermore, the flavonoid contents increased in both the OsmiR396‐sequestered and the *OsGRF8* overexpressing plants. By analysing 39 natural rice varieties, the elevated flavonoid contents were found to correlate with enhanced BPH resistance. Artificial applications of flavonoids to wild type (WT) plants also increased resistance to BPH. A BPH‐responsive flavanone 3‐hydroxylase (*OsF3H*) gene in the flavonoid biosynthetic pathway was proved to be directly regulated by OsGRF8. A genetic functional analysis of *OsF3H* revealed its positive role in mediating both the flavonoid contents and BPH resistance. And analysis of the genetic correlation between OsmiR396 and *OsF3H* showed that down‐regulation of *OsF3H* complemented the BPH resistance characteristic and simultaneously decreased the flavonoid contents of the MIM396 plants. Thus, we revealed a new BPH resistance mechanism mediated by the OsmiR396–OsGRF8–OsF3H–flavonoid pathway. Our study suggests potential applications of miRNAs in BPH resistance breeding.

## Introduction

Non‐protein‐coding RNAs are widespread and more common than previously thought (Morris and Mattick, 2014). Among them, microRNAs (miRNAs) of approximately 22 nucleotides in length regulate gene expression through antisense complementary binding to mRNAs at the post‐transcriptional level by their simple core mechanism (Ambros, 2001; Nelson *et al*., 2003). In plants, miRNAs function in development and determine physiological characteristics, and therefore have attracted attention as tools to improve crop plants (Tang and Chu, 2017; Wang and Wang, 2015). In addition, large numbers of miRNAs are involved in biotic and abiotic stress responses (Khraiwesh *et al*., 2012; Sunkar and Zhu, 2004), although the underlying mechanisms remain largely elusive. In *Arabidopsis*, miR393a is induced by bacterial flagellin and mediates resistance to bacteria through the auxin signalling pathway (Navarro *et al*., 2006). In rice (*Oryza sativa*), miR319 mediates response to rice ragged stunt virus by regulating the jasmonic acid (JA) biosynthetic and signalling pathways (Zhang *et al*., 2016). miR528 is sequestered by Argonaute 18 upon virus infection, activating its target gene and thereby promoting the accumulation of reactive oxygen species (ROS) for defence (Wu *et al*., 2017a). Plant small RNAs are delivered to pathogens by exosome‐like vesicles to inhibit their virulence through cross‐kingdom RNA interference (Cai *et al*., 2018). Nevertheless, the roles of plant miRNAs in plant–insect interactions and their underlying mechanisms have rarely been studied.

The functions of miR396 in plant development have largely been revealed. In *Arabidopsis*, miR396 is involved in leaf polarity, leaf development and root development (Das Gupta and Nath, 2015; Mecchia *et al*., 2013; Rodriguez *et al*., 2015). In rice, OsmiR396 regulates floral organogenesis and panicle development (Gao *et al*., 2015; Liu *et al*., 2014). miR396 is an important regulator in the reprogramming of root cells during nematode infections (Hewezi *et al*., 2012). However, whether and how miR396 functions in insect resistance is largely unknown.

A rice‐specific herbivore, the brown planthopper (BPH) is increasingly causing devastating yield losses throughout Asian planting areas (Cheng *et al*., 2013; Normile, 2008). Furthermore, BPH has evolved into different bio‐types that can adjust quickly and breakdown the resistance of rice species, making the pest more difficult to control. Currently, BPH control relies on extensive applications of environmentally unfriendly chemical insecticides. Therefore, there is an urgent need to identify different resistant resources for aggregated rice breeding (Zhang, 2007). In the past decade, great efforts have been made to identify plant endogenous resistance genes to BPH from different rice germplasms, and eight of them, *Bph14*,* Bph26*,* Bph3*,* Bph18*,* Bph29*,* BPH9*,* BPH32* and *BPH6*, have been successfully cloned (Du *et al*., 2009; Guo *et al*., 2018; Ji *et al*., 2016; Liu *et al*., 2015; Ren *et al*., 2016; Tamura *et al*., 2014; Wang *et al*., 2015; Zhao *et al*., 2016). In addition, reverse genetics has also contributed some gene resources (Guo *et al*., 2014; Zhou *et al*., 2009). However, our knowledge of the molecular mechanisms underlying these gene resources and further rice–BPH interactions is limited. Therefore, new genes are still desirable for theoretical studies and for rice cultivation to counteract the food security risk caused by BPH.

Flavonoids are a representative group of secondary metabolites that are widespread in plants and have protective functions against environmental stresses (Bharti *et al*., 2015; Nakabayashi and Saito, 2015; Pourcel *et al*., 2007). Flavonoid biogenesis and/or their regulatory pathway are involved in the resistance to various stresses, such as high and ultraviolet lights, low temperature, salinity, high sucrose and oxidation (Bharti *et al*., 2015; Ilk *et al*., 2015; Kusano *et al*., 2011; Lotkowska *et al*., 2015; Mahmood *et al*., 2016; Zhang *et al*., 2015). Flavonoids are powerful deoxidizers that relieve oxidative stress (Nakabayashi *et al*., 2014; Zhang *et al*., 2014), and respond quickly to different stresses (Dixon and Paiva, 1995). Plants may use flavonoids to deter the feeding, development and oviposition of herbivores (Onkokesung *et al*., 2014). Several flavonoids respond to insects, for example, vitexin inhibits *Spodoptera litura* larval growth (Aboshi *et al*., 2018) and schaftoside inhibits BPH growth by interacting with the native NICDK1 protein (Hao *et al*., 2018). However, how flavonoids are regulated in response to BPH attacks remains unknown.

To investigate the possible involvement of miRNAs in rice–BPH interactions, we performed miRNA sequencing analyses before and after BPH infestation and identified BPH‐induced OsmiR396. A functional analysis in three rice genetic backgrounds confirmed its negative regulatory effects on both BPH resistance and flavonoid content. Natural rice varieties exhibited enhanced BPH resistance as the flavonoid content increased. Through the functional analysis of OsmiR396, one target gene, *OsGRF8*, and one gene in flavonoid biosynthesis, *OsF3H*, were identified. A biochemical analysis of the direct regulation of OsGRF8 on *OsF3H* and a genetic correlation analysis between OsmiR396 and *OsF3H*, showed that OsmiR396–OsGRF8 modulated BPH resistance by directly regulating *OsF3H*. Thus, we revealed a new mechanism of BPH resistance involving the OsmiR396‐regulated flavonoid synthesis pathway.

## Results

### OsmiR396 was induced by BPH infestation

Because the miRNAs are so functionally diverse, we investigated whether they are involved in BPH resistance. The miRNA sequencing of rice seedlings at 4 h after BPH infestation was performed and compared with miRNA sequencing without infestation. There were 15 up‐regulated and 14 down‐regulated miRNAs in BPH‐infested compared with un‐infested plants (Table S1). In this study, we mainly focused on *OsmiR396b*.

**Figure 1:**
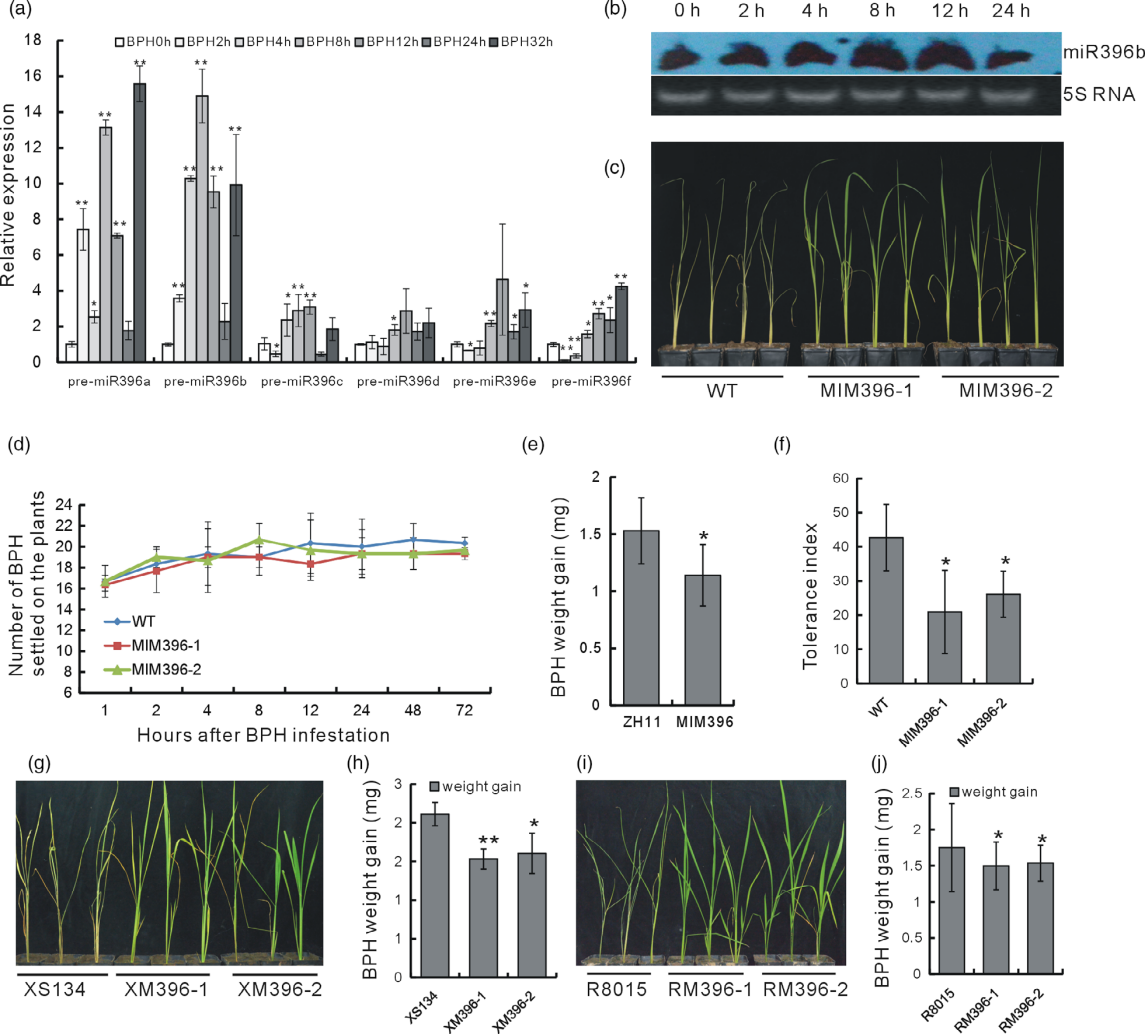
Detection of OsmiR396 expression after BPH feeding, and BPH resistance tests of the OsmiR396‐sequestered transgenic plants. (a) qRT–PCR analysis of the pre‐OsmiR396 transcripts after BPH infestation (*n* = 3). The expression level at 0 h was set as 1.0. (b) northern blot analysis of the expression levels of some OsmiR396s at different time points after BPH feeding using miR396b as the probe. (c) Individual tests to determine the BPH resistance of the MIM396 plants and the WT. (d) Analysis of the numbers of BPH that settled on the MIM396 and WT plants to indicate a choice tendency (*n* = 3). (e) Body weight gain of BPH feeding on the MIM396 and WT plants for 2 days (*n* = 10). (f) Tolerance indices of the MIM396 and WT plants after BPH feeding (*n* = 32). (g) Individual tests to determine the BPH resistance of the XM396 and WT (XS134) plants. (h) Body weight gains of the BPH feeding on the XM396 and WT (XS134) plants for 2 d (*n* = 10). (i) Individual tests to determine the BPH resistance of the RM396 plants and the WT (R8015). (j) Body weight gains of the BPH feeding on the XM396 and WT (R8015) plants for 2 days (*n* = 10). Asterisks in (a, e, f, h and g) represent significant differences determined by Student's *t*‐test at *P < 0.05 and **P < 0.01.

In rice, six genes, *OsmiR396a–f*, encode OsmiR396. To investigate the response of each *OsmiR396* gene to BPH infestation, we carried out a quantitative reverse transcriptase PCR (qRT–PCR) analysis of the pre‐OsmiR396 transcripts. Both pre‐OsmiR396a and pre‐OsmiR396b were induced by BPH infestation as early as 2 h, with peaks at 8 h, suggesting the possible involvement of these OsmiR396s in interactions with BPH (Figure 1a). Furthermore, compared with pre‐OsmiR396a, pre‐OsmiR396b showed continuous induction before 8 h. There appeared to be an induction rhythm for both pre‐OsmiR396a and pre‐OsmiR396b, because at 24 h, the expression levels of both miRNAs dropped to levels similar to those at 0 h, and promptly rose again at 32 h (Figure 1a). A northern blot analysis using OsmiR396b as the probe revealed that OsmiR396a/b was induced by BPH at 8 and 12 h (Figure 1b). Mature OsmiR396a and OsmiR396b shared the same sequence (http://www.mirbase.org/). Thus, at least some OsmiR396s were responsive to BPH. In particular, OsmiR396a and OsmiR396b were induced by BPH infestations.

### OsmiR396 negatively regulated rice resistance to BPH

To study the function of OsmiR396, we constructed a plasmid overexpressing OsmiR396 target mimicry (MIM396), intending to sequester the normal expression of native OsmiR396, and transformed it into the rice variety ZH11, which was used as WT. We obtained 35 transgenic MIM396 plants, and the positive plants were verified (Figure S1a). The transgenic plants showed no visible differences in plant height or tiller number as compared with the WT (Figure S1b). We tentatively selected the T1 generation of two transgenic lines and evaluated their responses to BPH using individual tests, and repeats were carried out in the following generations. After BPH infestation for 7 days, the MIM396 plants were still alive but the WT plants had died (Figure 1c). We then used one MIM396 line and carried out a small population analysis. The WT plants withered earlier than the MIM396 plants, indicating that the latter were more resistance than the former (Figure S2a). Additionally, the amount of honeydew excreted by BPH feeding on MIM396 plants decreased compared with that on WT, which further indicated the increased resistance of the MIM396 plants to BPH (Figure S2b) compared with WT.

Generally, there are three resistance strategies used by plants against insects: antixenosis to affect insect settling, colonization and oviposition; antibiosis to reduce the insect survival rate or feeding activity; and tolerance to withstand the damage by the insects (Jing *et al*., 2017). A host plant choice test revealed that there was no significant differences between the numbers of BPH that settled on the MIM396 and WT plants from 1 to 72 h after infestation (Figure 1d), which indicated that the resistance of the MIM396 plants to BPH was not a result of antixenosis. When we compared the body weight gain of the BPH after feeding on the genetically stable MIM396 plants for 2 days to that of BPH feeding on the WT, less weight was gained by the BPH feeding on the MIM396 plants (Figure 1e). Together with the decreased level of honeydew excretion by BPH feeding on the MIM396 plants (Figure S2b), we concluded that antibiosis contributed to the resistance of the MIM396 plants. Furthermore, we found that the tolerance index of the MIM396 plants was significantly lower than that of the WT (Figure 1f), indicating that the MIM396 plants also had an increased tolerance to BPH.

Next, to further verify the consequence of sequestering OsmiR396, we transformed the MIM396 plasmid into two other genetic rice backgrounds, R8015 and XS134, which are two main cultivated varieties in the Yangtze River Delta, and the corresponding transgenic plants were named as RM396 and XM396 respectively. Both the RM396 and the XM396 plants showed increased resistance levels to BPH, whether in individual tests (Figure 1g,i) or BPH weight gain tests (Figure 1h,j). Thus, sequestering OsmiR396 in different genetic rice backgrounds increased the plants’ resistance to BPH.

### OsmiR396 negatively regulated rice tolerance to salt stress

The OsmiR396‐sequestered plants showing resistance to BPH, a kind of biotic stress, were used to determine if they could mediate abiotic stress. The overexpression of OsmiR396c decreases tolerance to salt (Gao *et al*., 2010), indicating that OsmiR396 might negatively regulate salt tolerance. To test this hypothesis, we treated nutrition‐cultured MIM396 and WT plants with 100 mM NaCl (Figure S3a). After 7 days of treatment, the WT plants were nearly dead, whereas the MIM396 plants were still healthy (Figure S3b), and this contrast in their status was more obvious after recovering for 3 days (Figure S3c,d). Furthermore, an expression analysis verified that OsmiR396b could be induced by a salt treatment (Figure S3e). Thus, OsmiR396 negatively regulates the plants’ tolerance to salt, a kind of abiotic stress.

### Sequestering miR396 in both rice and *Arabidopsis* increased flavonoid contents

**Figure 2:**
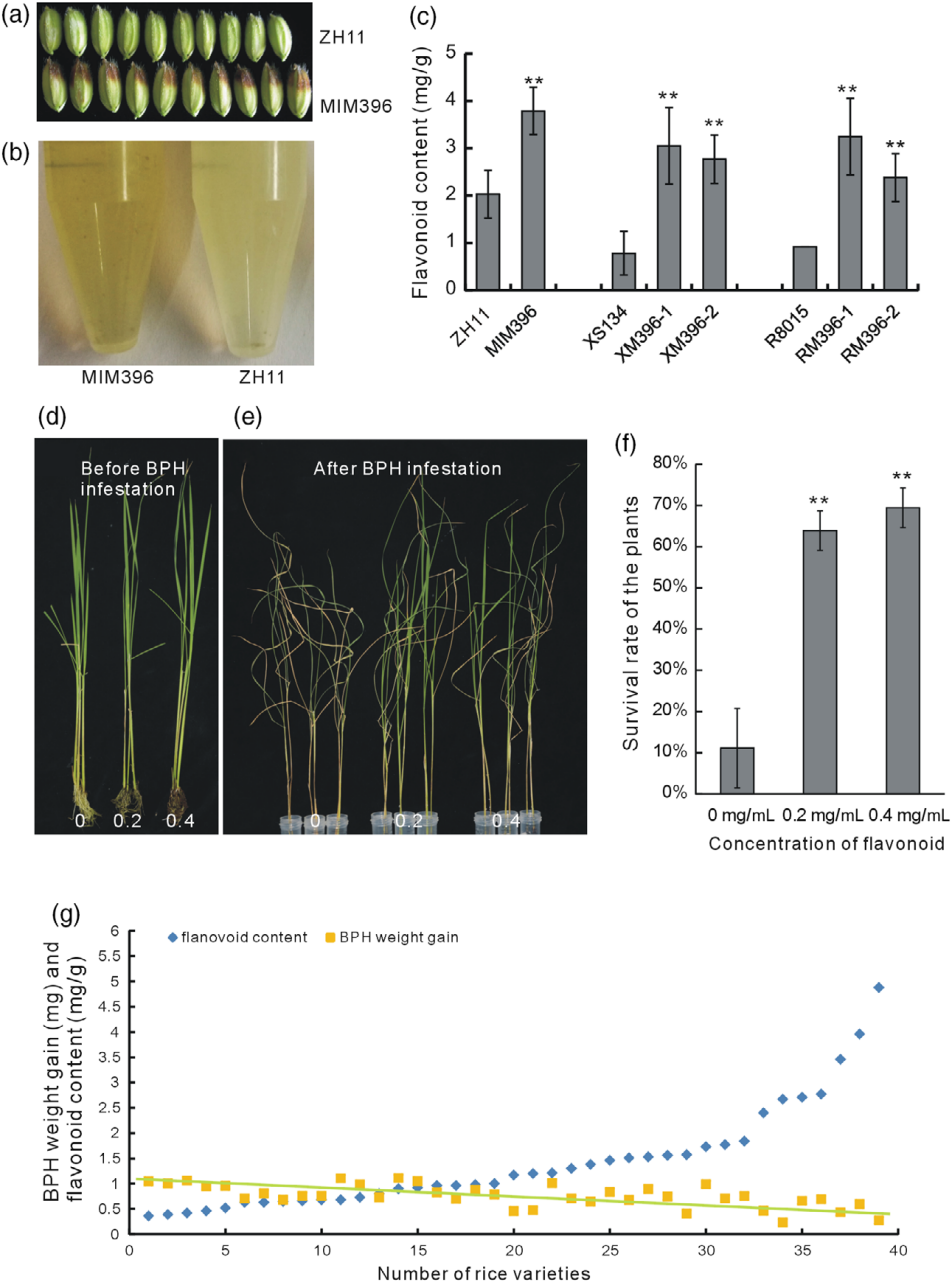
Detection of the correlation between flavonoid content and BPH resistance. (a) Rice grains of one MIM396 line and WT plants showing pigment deposition. (b) Flavonoids extracted from the MIM396 and WT plants showing the colour difference. (c) Quantitative determination of flavonoid contents in the MIM396 and ZH11 plants, XM396 and XS134 plants and RM396 and R8015 plants (*n* = 10). (d) Flavonoid‐treated WT plants before BPH feeding. The flavonoid concentrations were 0, 0.2 and 0.4 mg/mL. (e) Flavonoid‐treated WT plants after BPH feeding. (f) Statistical analysis of the survival rates of flavonoid‐treated WT plants after BPH feeding (*n* = 3). (g) Statistical analysis of the flavonoid contents in 39 natural rice varieties and the body weight gain of BPH feeding on the corresponding varieties for 2 days. The flavonoid contents of each variety are arranged in ascending order (*n* = 3), and BPH body weight gains are arranged according to the varieties (*n* = 10). The dashed line indicates the trend of BPH weight gain. Asterisks in (c) and (f) indicate significant differences determined by Student's *t*‐test at **P <* *0.01.

In the T0 generation of the MIM396 plants, the seed hulls of some lines were deeply coloured (Figure 2a), indicating possible changes in the flavonoid contents. Therefore, we extracted the total flavonoids from the hulls and found that they were indeed significantly increased, as indicated by the colour of the extract (Figure 2b) and by quantification (Figure 2c). Similarly, the flavonoid contents in the RM396 and XM396 plants also increased compared with their respective WT R8015 and XS134 plants (Figure 2c). Thus, sequestering OsmiR396 in different genetic backgrounds of rice increased the flavonoid contents.

Generally, miRNA functions are conserved among species. To verify the influence of OsmiR396 on the flavonoid contents, we genetically transformed the MIM396 plasmid into *Arabidopsis* Col‐0. The stems of the *Arabidopsis* transgenic plants (aMIM396) showed deeper colour than those of the WT (Figure S4a), and accordingly, the anthocyanin level was increased in the aMIM396 plants (Figure S4b,c). Thus, overexpression of MIM396 increased the flavonoid contents in both rice and *Arabidopsis*.

### Increased flavonoid content correlates with enhanced BPH resistance in rice

To determine whether the increased flavonoid contents in the OsmiR396‐sequestered plants accounted for their increased BPH resistance, we artificially treated the WT plants with flavonoids and fed them to the BPH. The flavonoids treatment did not result in any developmental changes to the plants (Figure 2d). When the plants were treated with 0.2 or 0.4 mg/mL flavonoid for 3 days, they showed greater resistance to BPH than the untreated plants (Figure 2e), and accordingly, the survival rate of the flavonoid‐treated plants increased (Figure 2f), indicating that the exogenously added flavonoids enhanced rice resistance to BPH. Therefore, the increased flavonoid contents in the OsmiR396‐sequestered plants may account for their increased BPH resistance.

To determine whether the flavonoid contents in rice was generally correlated with BPH resistance, we measured the flavonoid contents of 39 natural rice varieties and simultaneously analysed their resistance to BPH by determining the BPH weight gain after infestation. In general, the greater the flavonoid contents in different varieties, the lower the BPH weight gain, as shown in Figure 2g. Overall, the trend indicated that the flavonoid content was positively correlated with the resistance of rice to BPH.

### The OsmiR396–OsGRF8 module regulated flavonoid biosynthesis through a direct transcriptional regulation of *OsF3H*

**Figure 3:**
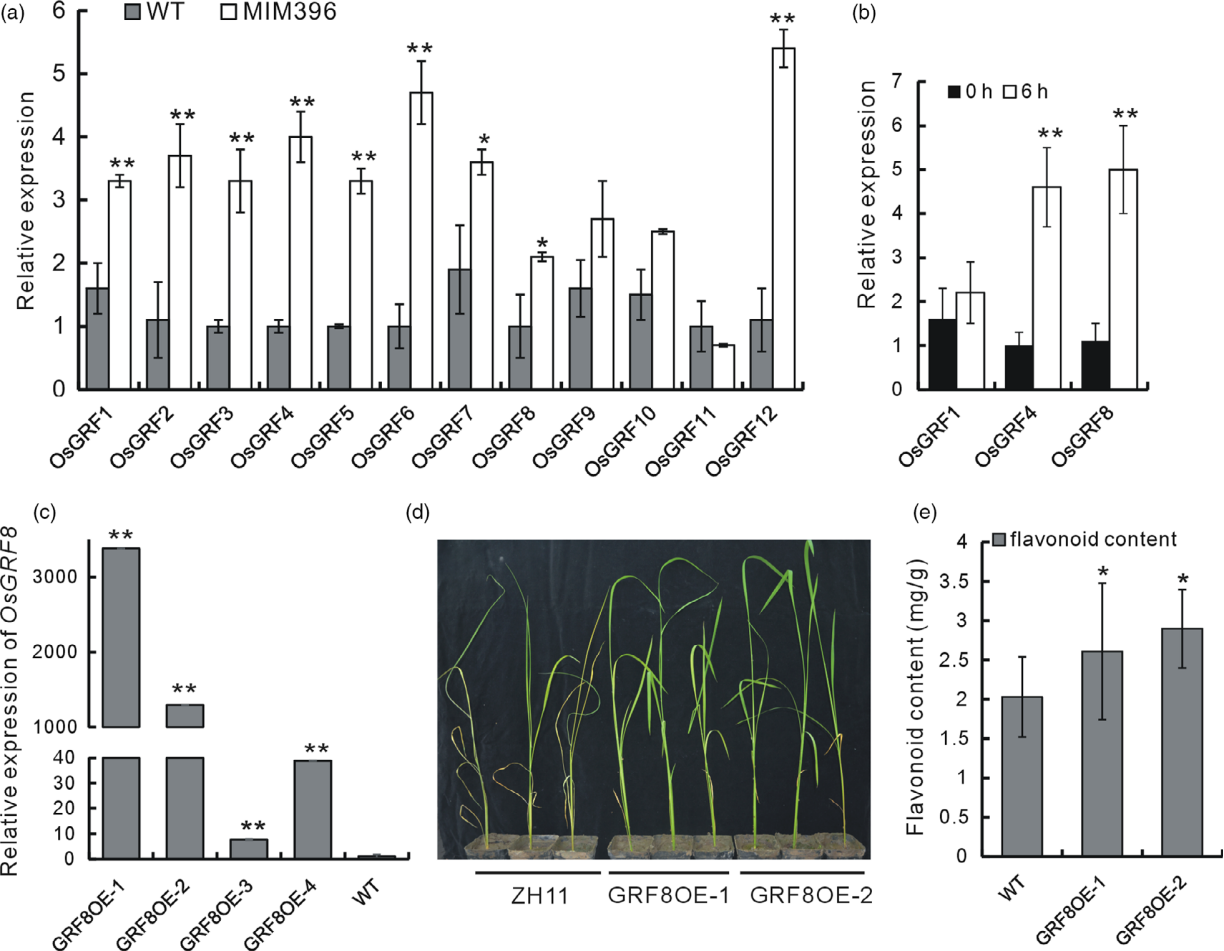
Expression characteristics of the *OsGRF* genes, and the function analysis of the *OsGRF8* gene. (a) Relative expression levels of the OsmiR396 target *OsGRF* genes in the MIM396 plants compared with in the WT (*n* = 3). (b) Relative expression of *OsGRF4* and *OsGRF8* upon BPH infestation (*n* = 3). (c) Analysis of the transcripts of *OsGRF8* in the GRF8 overexpressing (OE) transgenic plants (*n* = 3). (d) Individual analysis to determine the BPH resistance of the GRF8OE and WT plants. (e) Flavonoid contents in the GRF8OE and WT plants (*n* = 10).

There are 12 *OsGRF* genes that serve as OsmiR396 targets (Gao *et al*., 2015). In the MIM396 plants, the expression levels of most of the *OsGRF* genes were up‐regulated (Figure 3a). We further tested the response of each *OsGRF* gene to BPH infestation. *OsGRF4* and *OsGRF8* were up‐regulated at 6 h after BPH infestation (Figure 3b). We used *OsGRF8* for further functional analysis, and fused OsGRF8 with Green Fluorescent Protein (GFP) to construct overexpression transgenic lines (GRF8OE), in which *OsGRF8* was up‐regulated (Figure 3c). Using individual analysis, we tested two lines of GRF8OE plants and found them to be more resistant to BPH than the WT (Figure 3d). Moreover, the flavonoid contents in the GRF8OE plants increased (Figure 3e). On the basis of these analyses, we hypothesized that OsmiR396 mediates BPH resistance and flavonoid biosynthesis through *OsGRF8*.

**Figure 4:**
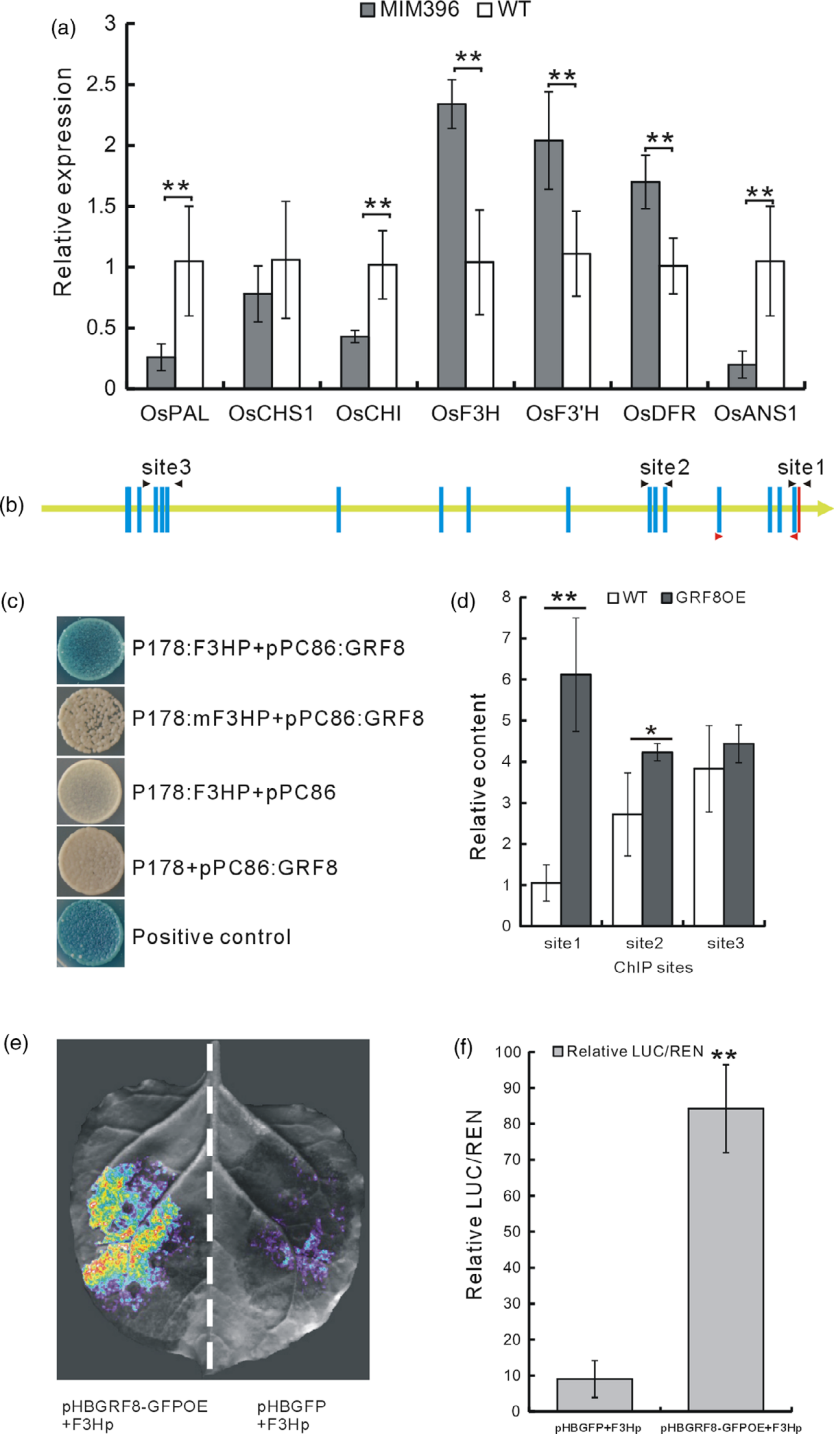
Analysis of the direct regulation of OsGRF8 on *OsF3H* gene. (a) qRT–PCR analysis of the transcripts of some genes in the flavonoid biosynthesis pathways of MIM396 and WT plants (*n* = 3). (b) Schematic representation of the 3‐kb *OsF3H* promoter showing the positions of the putative OsGRF8‐binding motifs and the relative sites for ChIP and yeast one‐hybrid analyses. The yellow line represents the *OsF3H* promoter with the gene coding direction indicated by the arrowhead; red bar indicates the ATG start code; blue bars indicate the predicted GRF‐binding motifs; sites 1, 2 and 3 indicated by the black arrowheads are the regions used for the ChIP analysis; the regions indicated by the red arrowheads were used for yeast one‐hybrid analysis. (c) Yeast one‐hybrid analysis of OsGRF8 and the *OsF3H* promoter. F3HP is the promoter region framed by the pair of red arrowheads in (b); and mF3HP is F3HP with mutations in the GRF‐binding motifs. (d) ChIP analysis of the *OsGRF8* overexpressing plants using the fused tag, GFP, as the antibody (*n* = 3). (e) Image of the Dual‐LUC assay. (f) LUC/REN ratio in the Dual‐LUC assay, indicating relative luciferase activity. The empty vector pHBGFP was used as the control in (e) and (f). Values are given as means ± SDs (*n* =3). Asterisks in (a, d and f) indicate significant differences determined by Student's *t*‐test at *P < 0.05 and **P < 0.01.

The increased flavonoid contents in the OsmiR396‐sequestered plants led us to hypothesize that OsmiR396 may regulate flavonoid biosynthesis. Consequently, we determined the expression levels of some flavonoid biosynthetic genes in the MIM396 plants and found that several genes were up‐regulated (Figure 4a). Among them, *OsF3H* (LOC_Os03g03034), had been induced by BPH feeding in a previous microarray analysis (Wang *et al*., 2012). Thus, *OsF3H* may mediate the increased flavonoid contents in the MIM396 plants.

The binding motif of GRF proteins is CGC(G)A(C)G(A) (Gao *et al*., 2015), and there are 17 such motifs in the 3‐kb promoter of the *OsF3H* gene (Figure 4b). To determine whether the OsGRF8 protein regulated *OsF3H* by binding to these motifs, we performed a yeast one‐hybrid assay and found that OsGRF8 could bind to the first four motifs in the *OsF3H* promoter. When these motifs were mutated, the binding disappeared (Figure 4c). We then carried out a chromatin immunoprecipitation (ChIP) assay using the GRF8OE plants and found that the GRF8‐fused GFP protein bound more fragments from the motif‐containing region close to the ATG start code in the *OsF3H* promoter compared with the other regions tested (Figure 4d). Furthermore, to validate the activation of *OsF3H* by OsGRF8, we carried out a Dual‐LUC assay in tobacco leaves, which revealed that OsGRF8 activated the expression of the *OsF3H* promoter, resulting in a greater LUC/REN value than the GFP control (Figure 4e,f). On the basis of these analyses, we concluded that the OsmiR396–OsGRF8 module activated the expression of *OsF3H* by directly binding of the OsGRF8 protein to the *OsF3H* promoter.

### *OsF3H* positively regulated both the BPH response characteristic and the flavonoid contents

**Figure 5:**
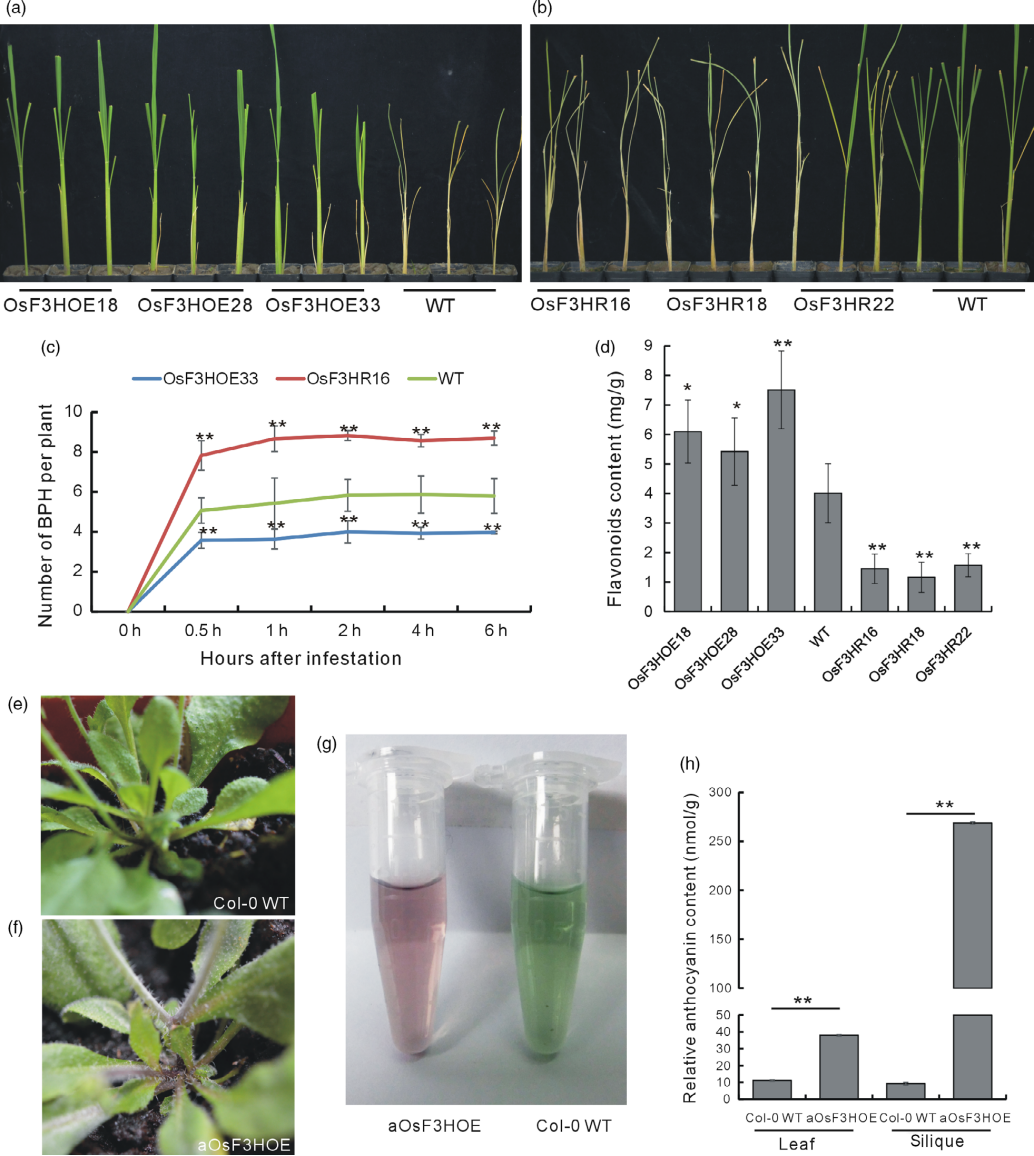
Biological functional analysis of the *OsF3H* gene. (a) Individual test to determine the BPH resistance of the OsF3HOE and WT plants. (b) Individual tests to determine the BPH resistance of the OsF3HR and WT plants. (c) Statistical analysis of the numbers of BPH settled on OsF3HOE33, OsF3HR16 and WT plants, indicating a choice tendency (*n* = 5). (d) Quantitative determination of flavonoid contents in the OsF3HOE, OsF3HRNAi and WT plants (*n* = 3). (e) Appearance of a Col‐0 WT *Arabidopsis* plant showing the stem colour. (f) Appearance of an aOsF3HOE *Arabidopsis* plant showing the stem colour. (g) Colour of the anthocyanin extracted from the aOsF3HOE and Col‐0 WT plants. (h) Quantitative determination of the anthocyanin contents in the leaves and siliques of the aOsF3HOE and Col‐0 WT plants (*n* = 3). Asterisks in (c, d and h) indicate significant differences determined by Student's *t*‐test at *P < 0.05 and **P < 0.01 compared with 0 h in (c) and WT in (d).

To determine whether *OsF3H* functioned in flavonoid biosynthesis and BPH resistance, we performed a genetic functional analysis of *OsF3H* using overexpression and double‐stranded RNA interference (RNAi). We obtained 36 *OsF3H* RNAi plants (OsF3HR) and selected three lines (OsF3HR16, OsF3HR18 and OsF3HR22) having the greatest degrees of *OsF3H* down‐regulation of *OsF3H* for further analyses (Figure S5a). Additionally, we obtained 32 *OsF3H* overexpression plants and selected three lines (OsF3HOE18, OsF3HOE28 and OsF3HOE33) with different degrees of *OsF3H* overexpression for further analyses (Figure S5b). Individual tests of BPH resistance showed that all three OsF3HOE lines were more resistant to BPH than the WT (Figure 5a), whereas all three lines of the OsF3HR plants were more susceptible to BPH feeding than the WT (Figure 5b). We further investigated the resistance mechanism and found that there were fewer BPH feeding on the OsF3HOE plants than on the WT, whereas BPH digested more of the OsF3HR plants than the WT, indicating that *OsF3H* regulated BPH resistance through a selection mechanism (Figure 5c).

We also measured the flavonoid contents in the *OsF3H* transgenic lines. In all of the OsF3HOE plants, the flavonoid contents were increased, whereas, in all of the OsF3HR plants, they were decreased (Figure 5d), which is consistent with the function of *OsF3H* in flavonoid biosynthesis. Furthermore, we overexpressed *OsF3H* in *Arabidopsis* Col‐0 and got aOsF3HOE plants. The aOsF3HOE plants showed increased pigment deposition (Figure 5f) compared with the WT (Figure 5e), and anthocyanin extracted from the aOsF3HOE plants was darker coloured than that of the WT (Figure 5g). The anthocyanin contents increased in the leaves and the siliques of the aOsF3HOE plants compared with in the WT (Figure 5h). Thus, overexpression of *OsF3H* in both rice and *Arabidopsis* increased the flavonoid contents. Hence, the *OsF3H* gene positively regulated both BPH resistance and flavonoid biosynthesis in rice.

### Complementation of BPH resistance in MIM396 plants through down‐regulation of *OsF3H*

**Figure 6:**
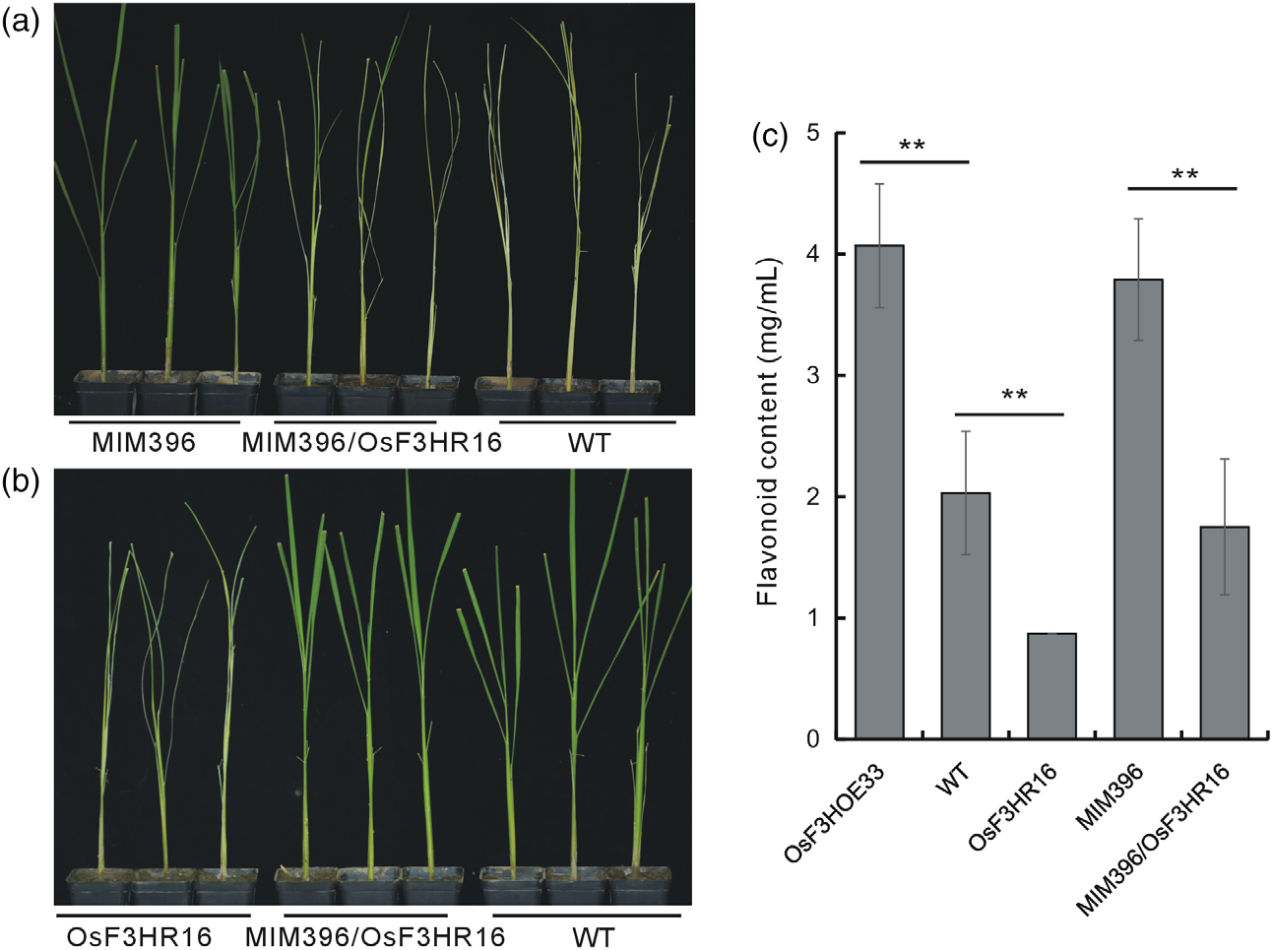
Functional analysis of a genetic cross between MIM396 and OsF3HR16 plants. (a) Individual tests to determine the BPH resistance of the MIM396/OsF3HR16 plants compared with those of the MIM396 and WT plants. (b) Individual tests to determine the BPH resistance of the MIM396/OsF3HR16 plants compared with those of the OsF3HR16 and WT plants. (c) Quantitative determination of the flavonoid contents in the MIM396/OsF3HR16, related transgenic and WT plants (*n* = 3). Asterisks indicate significant differences determined by Student's *t*‐test at **P <* *0.01.

To further verify the involvement of *OsF3H* in the BPH resistance pathway mediated by OsmiR396–OsGRF8, we crossed the OsF3HR16 plants with the MIM396 plants. Positive hybrids were tested for BPH resistance after verification at the genomic (Figure S6a) and mRNA (Figure S6b,c) levels. Compared with the MIM396 plants, the MIM396/OsF3HR16 plants were more susceptible to BPH feeding (Figure 6a), whereas, compared with the OsF3HR16 plants, they showed increased resistance to BPH (Figure 6b). This indicated that the resistance of the MIM396 plants was complemented by down‐regulation of the *OsF3H* gene. Furthermore, the flavonoid contents in the MIM396/OsF3HR16 plants were similar to those of the WT, indicating that the increased flavonoid contents in the MIM396 plants was also complemented by crossing with OsF3HR16 plants (Figure 6c). Thus, we concluded that down‐regulation of *OsF3H* decreased the BPH tolerance of the MIM396 plants by down regulating flavonoid biosynthesis.

### The salicylic acid (SA) signalling pathway was activated, whereas the JA signalling pathway was depressed in the MIM396 plants

Signalling molecules in several phytohormone pathways, such as JA and SA, are involved in the plant–insect interactions (Erb *et al*., 2012; Ling and Weilin, 2016). To investigate the possible signalling pathways involved in MIM396‐mediated resistance, we examined the transcript levels of genes involved in the SA‐ and JA‐dependent pathways during BPH infestation. The transcripts of *OsCoia* and *OsCoib* in the JA signalling pathway were suppressed in the MIM396 plants upon BPH infestation compared with in the WT (Figure S7a,b); whereas the *OsNPR1* gene in the SA signalling pathway was up‐regulated in the MIM396 plants upon BPH infestation (Figure S7c), which indicated that the MIM396 plants might mediate BPH resistance by activating the SA signalling pathway while suppressing the JA signalling pathway. Similarly, *Bph29* mediates BPH resistance by activating the SA signalling pathway, while inhibit the JA signalling pathway (Wang *et al*., 2015). *Bph9‐* and *Bph1‐* mediated BPH resistance by activating the SA signalling pathway (Du *et al*., 2009; Zhao *et al*., 2016).

## Discussion

miRNAs function extensively in plant development and determine physiological characteristics, and a great number of miRNAs are involved in various stress responses. However, little is known about the miRNAs mediating rice–BPH interactions, not to mention the detailed underlying mechanisms. Here, we revealed that many miRNAs are BPH‐responsive (Table S1), indicating their possible involvement in rice–BPH interactions. This corroborates the work of Wu *et al*. (Wu *et al*., 2017b), which showed that many miRNAs responded differentially to BPH in resistant and susceptible rice plants, indicating that miRNAs might mediate different pathways involved in the basal defence and specific resistance to BPH. We further revealed a detailed molecular mechanism mediated by one of the responsive miRNAs, OsmiR396, which was induced by BPH infestation (Figure 1a,b). Therefore, for the first time, the practical and detailed involvement of a miRNA was shown in rice–BPH interactions.

**Figure 7:**
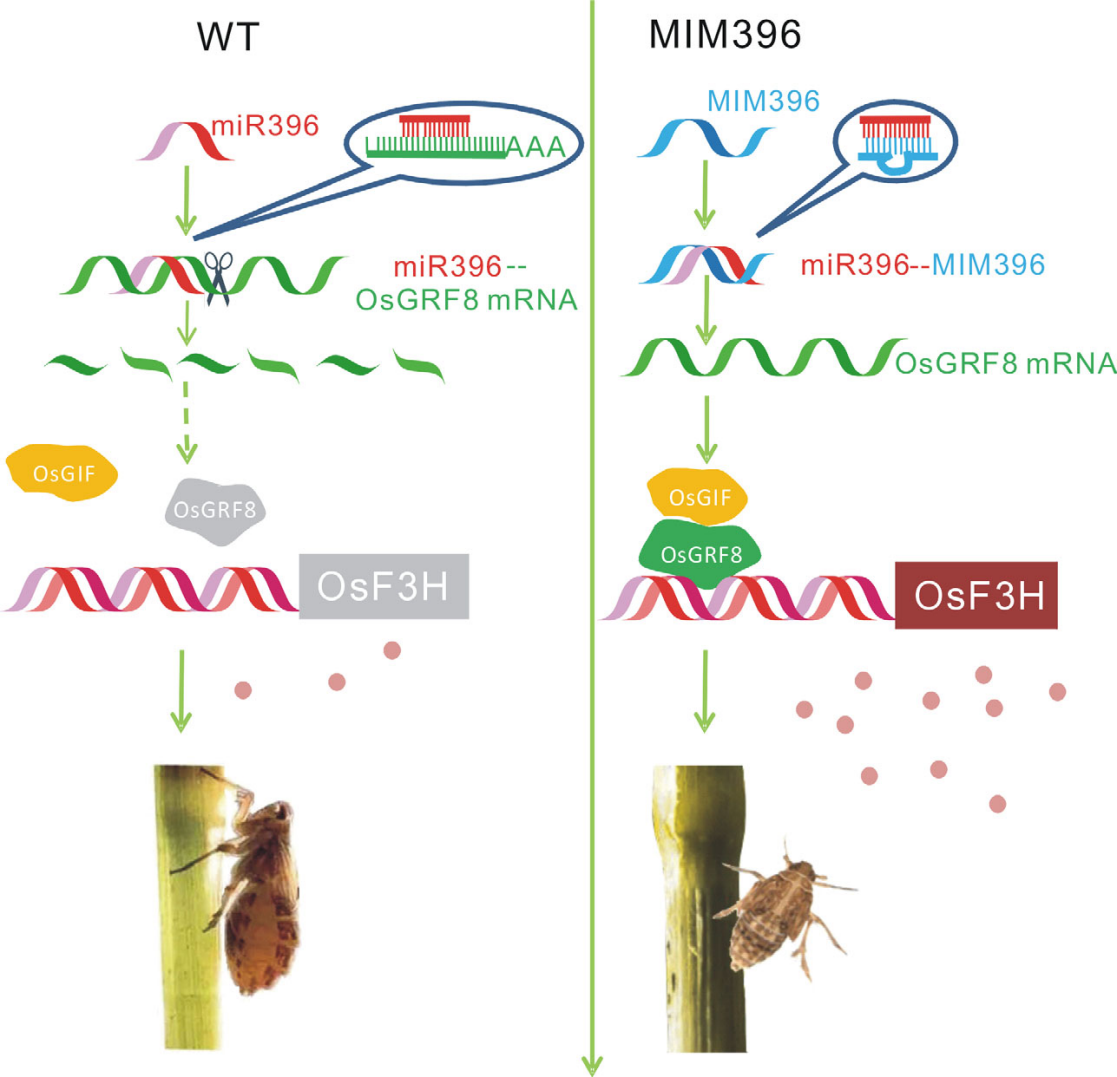
Schematic representation of the mechanism of the OsmiR396–OsGRF8–OsF3H–flavonoid pathway in BPH resistance. In WT plants, OsmiR396 cleaves *OsGRF8* transcripts and represses its expression, thereby suppressing the downstream flavonoid biogenesis, which is activated by OsGRF8. This results in plants being vulnerable to BPH attack. In MIM396 plants, OsmiR396 is sequestered by target mimicry miR396 (MIM396), resulting in *OsGRF8*′s expression, and OsGRF8 is available to up‐regulate the expression of *OsF3H*, which enhances flavonoid biogenesis and renders the MIM396 plants more resistant to BPH attack.

Plant hormones and signalling molecules play pivotal roles in plant immunity (Baxter *et al*., 2014; Pieterse *et al*., 2012; Yang *et al*., 2015), and the integration of miRNAs with these signalling pathways may be an important defence strategy employed by plants. The same might be true for the relationship of miRNAs and flavonoids. In this study, to elucidate the possible BPH resistance mechanism mediated by OsmiR396, we performed molecular, genetic and biochemical analyses, and proposed the involvement of an OsmiR396–OsGRF8–OsF3H–flavonoid pathway in response to BPH attacks. For the first time, we revealed a positive role for flavonoids in BPH resistance and its negative regulation by OsmiR396. In WT plants, OsmiR396 repressed *OsGRF8*, resulting in its inability to promote the expression of *OsF3H*. This resulted in low levels of flavonoids and rendered the WT plants more vulnerable to BPH attack (Figure 7). In the MIM396 plants, in which OsmiR396 was sequestered, the expression of *OsGRF8* was increased, resulting in the up‐regulation of *OsF3H* (Figures 3a and 4a). As a result, the flavonoid contents increased (Figure 2c), which rendered the OsmiR396‐sequestered plants more resistant to BPH infestation than the WT (Figure 7). The underlying mechanism responsible for the MIM396‐mediated resistance to BPH might involve flavonoids activating the SA signalling pathway while suppressing the JA signalling pathway (Figure S7). The positive role of SA in BPH resistance has been largely established (Ling and Weilin, 2016). The role of JA in BPH resistance is still controversial (Ling and Weilin, 2016), but increasing evidence supports the negative role of JA in BPH resistance. Because BPH is a piercing insect, the direct evidence is the increased susceptibility of rice plants to BPH after treatment with methyl jasmonate (data not shown). Furthermore, the *OsHI‐LOX* gene in the JA signalling pathway negatively regulates BPH resistance (Zhou *et al*., 2009), *BPH29* mediates resistance to BPH by depressing the JA signalling pathway (Wang *et al*., 2015), and brassinosteroids negatively regulate resistance to BPH by activating JA (Pan *et al*., 2018). Another possible mechanism downstream of OsmiR396‐mediated BPH resistance involves some flavonoids interacting directly with the NICKD1 protein in BPH and inhibiting their growth (Hao *et al*., 2018). This requires further investigation.

The BPH needs to use the rice resources to sustain growth and to propagate. In this study, we revealed that OsmiR396 functions diversely in rice. On one hand, the MIM396 plants were more resistant not only to BPH (Figure 1) but also to salt stress (Figure S3), indicating that OsmiR396 functions as a negative regulator of plant biotic and abiotic stresses. A BPH infestation up‐regulated the expression levels of some OsmiR396s (Figure 1a,b), and the induced OsmiR396s might function to break down the rice defence system, which would favour the invasion and growth of the BPH. Furthermore, the OsmiR396‐sequestered transgenic plants showed enlarged grains and improved seed quality (data not shown). These characteristics are consistent with the positive functions of its targets in grain size modulation. For example, *OsGRF4* positively regulates seed development and grain size (Che *et al*., 2015; Duan *et al*., 2015; Hu *et al*., 2015; Li *et al*., 2016). Thus, OsmiR396 negatively regulated grain size. Therefore, the induced OsmiR396 expression by BPH might also function to inhibit the reproductive growth of rice, rendering the rice plants more favourable to BPH growth by conserving more resources. At this point, OsmiR396 might provide an interaction target for the survival strategy of BPH. If this strategy is general or not would be clear along with the molecular mechanism underlying more and more BPH‐responsive miRNAs to be revealed.

In the OsmiR396–OsGRF8–OsF3H–flavonoid signalling pathway, MIM396 plants used both antibiotic and tolerance mechanisms to mediate BPH resistance (Figure 1e,f), whereas the *OsF3H* gene used a selection mechanism (Figure 5c). Because of the functional diversity of OsmiR396, one possible explanation is that in addition to *OsGRF8*, other targets of OsmiR396, such as *OsGRF4*, might mediate responses to BPH (Figure 3b). Therefore, more targets serving as transcriptional factors might regulate and recruit regulatory factors in other pathways to mediate BPH resistance. Furthermore, in the downstream signalling, in addition to the *OsF3H* gene, OsmiR396 might also regulate other genes in the flavonoid synthesis pathway, such as *OsPAL1*,* OsCHI*,* OsF3′H*,* OsDFR* and *OsANS*, to mediate BPH resistance (Figure 4a). These possibilities could influence the final resistance mechanisms integrated by OsmiR396.

One of the most common challenges for both conventional and modern crop improvement is that the promotion of a desirable trait is usually offset by the impairment of one or more other beneficial characteristics (Tang and Chu, 2017). In this study, OsmiR396 negatively regulated both biotic and abiotic stresses, as well as grain size, resulting in the OsmiR396‐sequestered transgenic plants not only having enlarged grains and improved seed quality, but also exhibiting enhanced resistance to both BPH and salt. Therefore, sequestering OsmiR396 in rice combined many breeding‐beneficial characteristics, and OsmiR396 may provide an ideal modulator for modern breeding.

## Experimental procedures

### Plant and BPH materials

One WT rice plants, and also the genetic background of most of the transgenic plants was ZH11 (*Oryza sativa* L. subsp. *japonica* cv. Zhonghua No.11). Rice cultivar varieties XS134 and R8015 (*Oryza sativa* L. subsp. *japonica*) were also used as host for transformation of MIM396 plasmid. Rice plants were grown in a paddy field or in pots in a greenhouse under standard growth conditions.

For *Arabidopsis thaliana,* the ecotype Columbia0 (Col‐0) was used as WT and transformed by MIM396 and F3HOE plasmids. Seeds were sown on Murashige and Skoog (MS) medium, cold‐treated for 3 days at 4 °C, and transferred to controlled environment cabinets under 8 h light/16 h dark conditions with a fluency rate of 120 μmol/s/m^2^ of white light (produced by cool‐white fluorescent lamps) at 22 °C.

The BPH population was originally obtained from rice fields in Shanghai, China and maintained on susceptible rice variety plants TN1 in a climate‐controlled room at 26 ± 2 °C, 12 h/12 h light/dark cycle and 80% relative humidity.

### miRNA sequencing analysis

WT rice plants of 3‐week‐old were individually infested with 12 second‐instar BPH nymphs that had been starved for 2 h, leaf sheaths were collected at 0 and 4 h with three biological samples each. Total RNA was extracted, purified and added adaptors to the 5′ and 3′ end using T4 ligase, amplified for miRNA library construction, and sequenced using Hiseq2500. Sequence analysis was carried out under the help of Genergy Biotechnology Company.

### Vector construction

Vector overexpressing OsmiR396 target mimicry (MIM396) was constructed previously (Gao *et al*., 2015).

For *OsGRF8* overexpression, the full‐length cDNA were amplified and cloned in fusion with GFP into the pHB vector under 35S promoter to form GRF8‐GFPOE plasmids and got GRF8OE plants after transformation. For *OsF3H* overexpression, full‐length cDNA was amplified by primers F3HOEF and F3HOER, and cloned into p1301‐35SNos vector. For RNAi of *OsF3H*, a 482 bp cDNA fragment of *OsF3H* gene was amplified and cloned into p1301RNAi vector in sense orientation using *Bam*HI and *Kpn*I, and antisense orientation using *Sac*I and *Spe*I.

Rice transformation was carried out by agrobacterium‐mediated method (Hiei *et al*., 1994); and flower dipping method was used for *Arabidopsis* transformation (Zhang *et al*., 2006).

### BPH resistance detection and measurements

Individual plant test was carried out at seedling stage using at least six replicates of each cultivar or line as described (Wang *et al*., 2012; Zhao *et al*., 2016). Each seedling about 3‐week old was infested with twelve second‐instar BPH nymphs. Plant status were observed daily, and 6–9 days later, the plants were scored as susceptible (dead) or resistant (alive).

For small population analysis, about 100 plants from each line were planted in a small pot in the mud for 1 month, and feed to BPH population in appropriately 10–15 first‐instar nymphs per plant, and then surveyed daily in the following week.

The BPH weight gain, honeydew measurement and host choice test were performed as described (Du *et al*., 2009; Wang *et al*., 2012; Zhao *et al*., 2016). The tolerance test was carried out as described by Cohen *et al*. (Cohen *et al*., 1997).

For BPH resistance analysis of the flavonoid‐treated plants, WT seeds were sterilized and cultured in 1/2 MS liquid medium. At about 3 weeks, four seedlings were treated with 20 mL of culture medium applied with 0, 0.2 and 0.4 mg/mL flavonoid, respectively, and then 40 first‐instar BPH nymphs were applied to the seedlings. Each treatment was in triplicates and three repeats were carried out. Plant damage levels were observed daily, and the plant survival rate was measured 5 days after infestation.

### NaCl (salt) treatment

For salt treatment, 2‐week‐old seedlings of the MIM396 and WT plants were transferred to plastic containers containing 1/2 MS liquid medium with 100 mm NaCl for 1 week, and recovered by water for 3 days (*n* = 32). Experiments were carried out in three repeats.

### Anthocyanin and flavonoid content measurements

In this study, anthocyanin content was measured for *Arabidopsis* plants as previously described (Gou *et al*., 2011) and flavonoid content was measured for rice plants accordingly to Sun *et al*. with modification (Sun *et al*., 2016). 1 g powder milled from fresh leaves was taken for extraction with 10 mL 50% ethanol for 1 h and repeated 2–3 times, and made up to 50 mL. 0.5 mL of supernatant was taken out from each extract and made up to 5 mL using 30% ethanol, and 5 min later, added 0.5 m NaNO_2_ 0.3 mL, 0.3 m AlCl_3_ 0.3 mL and vortexed, and 6 min later, added 1 m NaOH 2 mL, and made up to 10 mL using distilled water, and 1 min later, absorbance was read at 510 nm against a blank sample. The flavonoid content was represented as a percentage, using a standard Rutin curve made beforehand. Each measurement was repeated three times.

### RNA isolation and qRT–PCR analysis

Total RNAs were extracted using TRIzol (Life technologies) and reverse transcribed using the First Strand cDNA Synthesis Kit (Toyobo). qRT–PCR was performed with the SYBR Green Real‐time PCR Master Mix Kit (Toyobo), and *actin* gene was used as an internal control. Each sample was performed in triplicate and the mean value of technical replicates was recorded for each biological replicate. Data from three biological samples were collected, and the mean value with standard error was plotted.

For qRT–PCR analysis of genes to BPH response, 3‐week‐old rice seedlings were individually infested with 12 second‐instar BPH nymphs, and leaf sheaths were collected after 0, 2, 4, 8, 12 and 24 h for RNA extraction.

For qRT–PCR analysis of genes to salt response, 3‐week‐old rice seedlings were treatment with 100 mm NaCl and leaves were collected at 0, 1,2 4, 8, 12 and 24 h for RNA extraction.

### Yeast‐one‐hybrid assays

The full‐length cDNAs of *OsGRF8* were amplified and fused with the activation‐domain (AD) of pPC86 vector. Fragments containing four putative GRF‐binding motifs in *OsF3H* promoter were amplified and fused into vector p178 at the *Xho*I site to get p178:F3HP. The fragments with mutant GRF‐binding motifs were constructed based on the p178:F3HP plasmid, mutants in the two outer motifs were introduced in the primers, and mutants in the two inner motifs were introduced through overlapping PCR.

The respective p178 and pPC86 constructs were transformed into the yeast strain EGY48 and grew on SD selective medium (SD‐His‐Leu) and observed on Chromogenic medium. Void plasmid pPC86 and p178 constructs were used as negative controls.

### miRNA northern blot analysis

miRNA northern blotting was carried out as described with modifications (Wang *et al*., 2010). The OsmiR396b probe was synthesized with 3′‐End Biotin. The blots were incubated at 42 °C for 30 min in the Hybridization Buffer (Ambion). And 50–80 pm probes were added in the hybridization buffer to incubate for one night.

### ChIP analysis

Immunoprecipitation of DNA associated with modified histones was carried out according to the EpiQuik™ Plant ChIP Kit (Epigentek). Rice young panicles were cross‐linked in 1% formaldehyde, quenched cross‐linking and washed twice in deionized water. The resulting extract was sonicated to fragment chromatin (4 × 10 s burst/5 min rest, 280 v) and centrifuged for 10 min at 17 500 ***g***. Binding antibody to the assay plate and chromatin was immune‐precipitated with GFP antibody. At last, immune precipitated sample and whole‐cell extract (input) were incubated at 65 °C to reverse cross‐linked DNA, and ethanol precipitation to elute purified DNA. ChIP DNA and input were subjected to qRT–PCR using the primers designed to amplify a sequence in the promoter, a sequence in the coding region was used as control.

All the primer sequences used in this study were listed in Table S2.

### Dual luciferase (LUC) analysis

The plasmid pHBGRF8‐GFPOE was transformed into *Agrobacterium tumefaciens* strain GV3101 to act as effector. The reporter was constructed by inserting the promoter of *OsF3H* into pGreenII 0800‐LUC vector (Hellens *et al*., 2005) and subsequently co‐transformed with the helper plasmid pSoup19 into GV3101, with pHBGFP as negative control. Overnight *A. tumefaciens* cultures were collected by centrifugation and re‐suspended in MS medium to OD600 = 0.6, and incubated at RT for 3 h. The reporter and effectors strains were mixed at the ratio of 1:1 and infiltrated into tobacco (*Nicotiana benthamiana*) leaves and the negative control was infiltrated into the opposite position on the same leaves. Leaves were collected after 3 days (long day/white light) and infiltrated with 150 μg/mL luciferin solution; images were captured using a CCD camera 5 min later and quantification was performed using Dual‐Luciferase Reporter Assay System (Promega, Madison, WI). Three biological repeats were measured for each sample.

## Accession numbers

The accession numbers used in this study were listed in Table S2.

## Conflict of interest

The authors declare no conflict of interests.

## Supporting information


**Table S1** BPH‐responsive miRNAs identified by miRNA sequencing analysis.
**Table S2** Primer sequences and accession numbers used in this study.
**Figure S1** Verification and characterization of the MIM396 plants.
**Figure S2** BPH resistance of the MIM396 plants in small population test (a) and in test of the contents of honeydew excreted by the BPH after feeding for 2 days (b).
**Figure S3** Tolerance of the MIM396 plants to 100 mm NaCl.
**Figure S4** Analysis of anthocyanin contents in the aMIM396 *Arabidopsis* plants.
**Figure S5** qRT–PCR analysis of the transcript of *OsF3H* gene in OsF3HR (a) and OsF3HOE (b) plants compared with in the WT respectively.
**Figure S6** Molecular analysis of the genetic cross between MIM396 and OsF3HR16 plants.
**Figure S7** qRT–PCR analysis of the transcripts of some signalling genes in JA and SA pathway during BPH infestation in the MIM396 plants compared with in the WT.
