# Supplementary material for: The OsmiR396–OsGRF8–OsF3H‐flavonoid pathway mediates resistance to the brown planthopper in rice (Oryza sativa)
Source: Plant Biotechnol J. 2019 Mar 13;17(8):1657–69. doi: 10.1111/pbi.13091 (PMC6662109; doi:10.1111/pbi.13091)
Supplement: Supplementary file 1 — Table S1 BPH‐responsive miRNAs identified by miRNA sequencing analysis. Table S2 Primer sequences and accession numbers used in this study. Figure S1 Verification and characterization of the MIM396 plants. Figure S2 BPH resistance of the MIM396 plants in small population test (a) and in test of the contents of honeydew excreted by the BPH after feeding for 2 days (b). Figure S3 Tolerance of the MIM396 plants to 100 mm NaCl. Figure S4 Analysis of anthocyanin contents in the aMIM396 Arabidopsis plants. Figure S5 qRT–PCR analysis of the transcript of OsF3H gene in OsF3HR (a) and OsF3HOE (b) plants compared with in the WT respectively. Figure S6 Molecular analysis of the genetic cross between MIM396 and OsF3HR16 plants. Figure S7 qRT–PCR analysis of the transcripts of some signalling genes in JA and SA pathway during BPH infestation in the MIM396 plants compared with in the WT. [file PBI-17-1657-s001.docx]

**Supplementary Material**

**The miR396**–**GRF8**–**F3H-flavonoid pathway mediates resistance to the brown planthopper in rice (*Oryza* *sativa*)**

Zhengyan Dai^1, †^, Jiang Tan^1, †^, Cong Zhou^2^, Xiaofang Yang^1,3^, Fang Yang^2^, Shijuan Zhang^4^, Shichen Sun^5^, Xuexia Miao^1*^, Zhenying Shi^1*^

^1^Key Laboratory of Insect Developmental and Evolutionary Biology, Institute of Plant Physiology and Ecology, Shanghai Institutes for Biological Sciences, Chinese Academy of Sciences, Shanghai, 200032, China

^2^State Key Laboratory of Hybrid Rice, College of Life Sciences, Wuhan University, Wuhan, 430072, China

^3^University of Chinese Academy of Sciences, Shanghai, 200032, China

^4^Shandong Province Key Laboratory of Life-Organic Analysis, Qufu Normal University, Qufu, PR China

^5^Institute of Crop Cultivation and Tillage, Heilongjiang Academy of Agricultural Sciences &Northern Japonica Rice Molecular Breeding Joint Research Center, Chinese Academy of Sciences, Haerbin, 150086, China.

^†^ These authors contributed equally to this work

***Corresponding authors.**

E-mail: [xxm@sibs.ac.cn](mailto:xxm@sibs.ac.cn); [zyshi@sibs.ac.cn](mailto:zyshi@sibs.ac.cn).

**Table S1.** BPH-responsive miRNAs identified by miRNA sequencing analysis.

| miRNAs | fold change |  | miRNAs | fold change |
| --- | --- | --- | --- | --- |
| osa-miR399b | 2.95 |  | osa-miR1846e | -3.61 |
| osa-miR1861d | 2.50 |  | osa-miR7693 | -3.44 |
| osa-miR398b | 2.33 |  | osa-miR156b | -2.97 |
| osa-miR408 | 2.20 |  | osa-miR1850 | -2.68 |
| osa-miR399d | 2.15 |  | osa-miR166l | -2.66 |
| osa-miR5072 | 2.10 |  | osa-miR169a | -2.57 |
| osa-miR528 | 2.10 |  | osa-miR1428f | -2.22 |
| osa-miR396b | 1.98 |  | osa-miR2867 | -2.05 |
| osa-miR1861g | 1.94 |  | osa-miR169m | -1.93 |
| osa-miR1861c | 1.86 |  | osa-miR1857 | -1.92 |
| osa-miR1861k | 1.78 |  | osa-miR5504 | -1.84 |
| osa-miR166j | 1.63 |  | osa-miR169e | -1.70 |
| osa-miR169i | 1.55 |  | osa-miR169f | -1.58 |
| osa-miR399j | 1.52 |  | osa-miR169r | -1.43 |
| osa-miR5076 | 1.37 |  |  |  |

**Table S2.** Primer sequences and accession numbers used in this study.

| Primer names | Sequences (5’-3’) | usage | Corresponding accession NO. |
| --- | --- | --- | --- |
| IPSF | GTGGATCCaagaaaaatggccatcccctagc | MIM396 construction | AT1G29265 |
| IPSR | CTGGAGCTCgaggaattcactataaagagaatcg | MIM396 construction | AT1G29265 |
| ActinrealF | CAGCCACACTGTCCCCATCTA | qRT–PCR | LOC*_*Os03g61970 |
| ActinrealR | AGCAAGGTCGAGACGAAGGA | qRT–PCR | LOC*_*Os03g61970 |
| MIM396b-I | cgaagctUUCCACAGCUUtagaUCUUGAACUGtttctagagggagataa | MIM396 construction |  |
| MIM396b-II | cctctagaaaCAGTTCAAGATCTAAAGCTGTGGAAagcttcggttcccctcg | MIM396 construction |  |
| GRF1OEF | CCCAAGCTTATGATGATGATGAGCGGTCGCC | pHB-OsGRF1 | LOC_Os02g53690 |
| GRF1OER | CGGACTAGTATCATCATTGTGGTAGCGGGAG | pHB-OsGRF1 | LOC_Os02g53690 |
| GRF4OEF | CCCAAGCTTatggcgatgccgtatgcctcc | pHB-OsGRF4 | LOC_Os02g47280 |
| GRF4OER | CGGACTAGTGTCACCATTAGTTGATC | pHB-OsGRF4 | LOC_Os02g47280 |
| GRF8OEF | aaatcaccagtctctctctcAAGCTTATGCTGAGCTCTTGTGGTGGCCA | pHB-OsGRF8 | LOC_Os11g35030 |
| GRF8OER | TCCTCGCCCTTGCTCACCATACTAGTGAGAAGTGTTGGGACAATATGA | pHB-OsGRF8 | LOC_Os11g35030 |
| GRF1realF | AAAGAGGACGACGATGAGAAAGAG | qRT–PCR | LOC_Os02g53690 |
| GRF1realR | GCCCAGGAGGAAGCAGTG | qRT–PCR | LOC_Os02g53690 |
| GRF2realF | TACGGACGGCAAGAAGTG | qRT–PCR | LOC_Os06g10310 |
| GRF2realR | GGCATTTCCACAGGCTTTC | qRT–PCR | LOC_Os02g10310 |
| GRF3realF | CAATGCTGCGTCTTACTC | qRT–PCR | LOC_Os04g51190 |
| GRF3realR | AATGTGGAGGTCTGAGAAG | qRT–PCR | LOC_Os04g51190 |
| GRF4realF | CATCTGTTGTCGGTTCTG | qRT–PCR | LOC_Os02g47280 |
| GRF4realR | GCAATAGCAGGGTAAAGAG | qRT–PCR | LOC_Os02g47280 |
| GRF5realF | TTCTTCTCAGGAGCATCAG | qRT–PCR | LOC_Os06g02560 |
| GRF5realR | GTTCAAGGTGGGAGTAGG | qRT–PCR | LOC_Os06g02560 |
| GRF6realF | CCTCGCTATCAACCATCAG | qRT–PCR | LOC_Os03g51970 |
| GRF6realR | GCACTTGTTCACTCTCATTATG | qRT–PCR | LOC_Os03g51970 |
| GRF7realF | TTGGATCAGGTGGCTATC | qRT–PCR | LOC_Os12g29980 |
| GRF7realR | TTGTGTTGGTGTGAATGG | qRT–PCR | LOC_Os12g29980 |
| GRF8realF | GCAAGAGCAAGAGCAAGATG | qRT–PCR | LOC_Os11g35030 |
| GRF8realR | GCAAGAGCAAGAGCAAGATG | qRT–PCR | LOC_Os11g35030 |
| GRF9realF | GCTCATTGCCATCTTCTGTC | qRT–PCR | LOC_Os03g47140 |
| GRF9realR | GTTCGCCATTGTCCTGTTC | qRT–PCR | LOC_Os03g47140 |
| GRF10realF | TGCTCATCTACCGCTACTTC | qRT–PCR | LOC_Os02g45570 |
| GRF10realR | CGACGCTCTTCCAGATGG | qRT–PCR | LOC_Os02g45570 |
| GRF11realF | TGCCTACTCATCTCGTCTTC | qRT–PCR | LOC_Os07g28430 |
| GRF11realR | GTTCTGGTTCTGGGTTCTTTC | qRT–PCR | LOC_Os07g28430 |
| GRF12realF | TCAAGAAAGCCTATGGAAGCCTCTG | qRT–PCR | LOC_Os04g48510 |
| GRF12realR | TGGCGACGGTGTGGAGTG | qRT–PCR | LOC_Os04g48510 |
| GRF8EcoRI2F | GGGTACCgagaagtgttgggacaatatga | For pPC86 construction | LOC_Os11g35030 |
| GRF8SpeIR | gtggtcgccatcgAtcTagCaaCcaAgtAgaTggccgaaa | For pPC86 construction | LOC_Os11g35030 |
| F3HYOHwF | GGCTCGAGGCTCATTAGATTCATCTCGCAA | For p178:F3HP | LOC_Os03g03034 |
| F3HYOHwR | GGCTCGAGATGCCGCGGTACACACA |  | LOC_Os03g03034 |
| F3HYOHmF | GGCTCGAGGCTCATTAGATTCATCTaaattattag | For p178:mF3HP motif 1 mutation | LOC_Os03g03034 |
| F3HYOHmR | GGCTCGAGAaaaaaCGGTACACACA | For p178:mF3HP motif 4 mutation | LOC_Os03g03034 |
| F3HYOH2mR | aggcgcgacgaaaaaccagcggagaa | For p178:mF3HP motif 2 mutation using overlapping PCR | LOC_Os03g03034 |
| F3HYOH2mF | ttctccgctggtttttcgtcgcgcct |  | LOC_Os03g03034 |
| F3HYOH3mF | cccctctccccctttttcctgactcgactt | For p178:mF3HP motif 3 mutation using overlapping PCR | LOC_Os03g03034 |
| F3HYOH3mR | aagtcgagtcaggaaaaagggggagagggg |  | LOC_Os03g03034 |
| OsPALF | TACACCGACCACCTCATC | qRT–PCR |  |
| OsPALR | GCTGCCTTCCAGTATGTG | qRT–PCR |  |
| OsCHS1F | ACTGCGTGTACCAGGCCGACTA | qRT–PCR | LOC_Os11g32650 |
| OsCHS1R | TGGTGCAGAAGACGAGGTGGGT | qRT–PCR | LOC_Os11g32650 |
| OsCHIF | AAGGAGGCCTTCAAGCCCCACA | qRT–PCR | LOC_Os03g60509 |
| OsCHIR | CCGTGCTCGCCGATGATGGAAT | qRT–PCR | LOC_Os03g60509 |
| OsF3’HF | GACAAGCCGCACCACACCATGT | qRT–PCR | LOC_Os10g17260 |
| OsF3’HR | AGGTTCCTCACCATGAGCGCGA | qRT–PCR | LOC_Os10g17260 |
| OsDFRF | TCAAGCCCACCGTGGAAGGGAT | qRT–PCR | LOC_Os01g44260 |
| OsDFRR | TCGATGTCGCTCCAGTCGTCGT | qRT–PCR | LOC_Os01g44260 |
| OsANS1F | AGTGGGGCGTCATACACATCGC | qRT–PCR | LOC_Os01g27490 |
| OsANS1R | AGAGGATGGCGAGCAGCTTGGA | qRT–PCR | LOC_Os01g27490 |
| F3HPLUCF | gggtaccTCTGATGTGACACGCCAAAA | Luciferase analyis | LOC_Os03g03034 |
| F3HPLUCF | gggatcCGCGGTACACACACACAACTTG | Luciferase analyis | LOC_Os03g03034 |
| F3HOEF | cgggatccGCCAAGTTGTGTGTGTGTACC | OsF3HOE | LOC_Os03g03034 |
| F3HOER | ggggtaccCGAGAAACTACGGGGGTGTT | OsF3HOE | LOC_Os03g03034 |
| F3HRNAiF | gggactagtggtaccGAGCGATATCCGAGAGCCTG | OsF3HRNAi | LOC_Os03g03034 |
| F3HRNAiR | gggagctcggatccTTGGTCGAGGTTTCTGCTCC | OsF3HRNA | LOC_Os03g03034 |
| F3H-RT-F | CGCTACCTCCCTGATTGG | qRT-PCR | LOC_Os03g03034 |
| F3H-RT-R | ACCTTCTTGATGTAGTCCTGTTC | qRT-PCR | LOC_Os03g03034 |
| ChIPsite1F | GGTGCGTGCCAAGTTGTG | ChIP analysis | LOC_Os03g03034 |
| ChIPsite1R | GCAGCGTGTCGTGGTCTG | ChIP analysis | LOC_Os03g03034 |
| ChIPsite2F | ATTCCGAACCACACACTG | ChIP analysis | LOC_Os03g03034 |
| ChIPsite2R | CCTTACTCCAACGCTACG | ChIP analysis | LOC_Os03g03034 |
| ChIPsite4F | CGTCAAGAGGAGGAACTG | ChIP analysis | LOC_Os03g03034 |
| ChIPsite4R | ACTGAATACAAGCCGTAGG | ChIP analysis | LOC_Os03g03034 |
| SacI | CCGAGTTGGTCAAAGGAAAA | Check OsF3HRNAi plants |  |
| 396aF | CTTTGTGATCTTCCACAGCTT | qRT–PCR | miR396a |
| 396aR | GCAACCGATCGAGTCTAACTA | qRT–PCR | miR396a |
| 396bF | CTTTGTGGTCTTCCACAGCTT | qRT–PCR | miR396b |
| 396bR | TGCAAGCAAAATCCACACATA | qRT–PCR | miR396b |
| 396cF | TGCCATGCCTTTCCACAGCTT | qRT–PCR | miR396c |
| 396cR | GCACTCCTCTCCCTATAAGCT | qRT–PCR | miR396c |
| 396dF | GATGTGCGGGCATGCTTTC | qRT–PCR | miR396d |
| 396dR | TGGAGGCCACCGACCACA | qRT–PCR | miR396d |
| 396eF | GGGCATGCTTTCCACAGGC | qRT–PCR | miR396e |
| 396eR | AGACAGAACGGGGAGCTCGAC | qRT–PCR | miR396e |
| 396fF | GCCATGCTCTCCACAGGC | qRT–PCR | miR396f |
| 396fR | GATGACCTAATTCAGATCAGATC | qRT–PCR | miR396f |
| OsNPR1F | GAGGACAAGGAGGAGAATG | qRT–PCR | LOC_Os01g09800 |
| OsNPR1R | ACAGAAGGCAACCATCAG | qRT–PCR | LOC_Os01g09800 |
| OsCoiaF | ATGACTGAACTCAAAGTGGCG | qRT–PCR | [LOC_Os01g63420](http://rice.plantbiology.msu.edu/cgi-bin/gbrowse/rice?name=LOC_Os01g63420) |
| OsCoiaFR | TCGTAGAATGCTCCTCCAGC | qRT–PCR | [LOC_Os01g63420](http://rice.plantbiology.msu.edu/cgi-bin/gbrowse/rice?name=LOC_Os01g63420) |
| OsCoibF | CCTGGAGCTTCTCGCAAAGA | qRT–PCR | [LOC_Os05g37690](http://rice.plantbiology.msu.edu/cgi-bin/gbrowse/rice?name=LOC_Os05g37690) |
| OsCoibR | GTGAGCTCCCCTTGCTCAAT | qRT–PCR | [LOC_Os05g37690](http://rice.plantbiology.msu.edu/cgi-bin/gbrowse/rice?name=LOC_Os05g37690) |


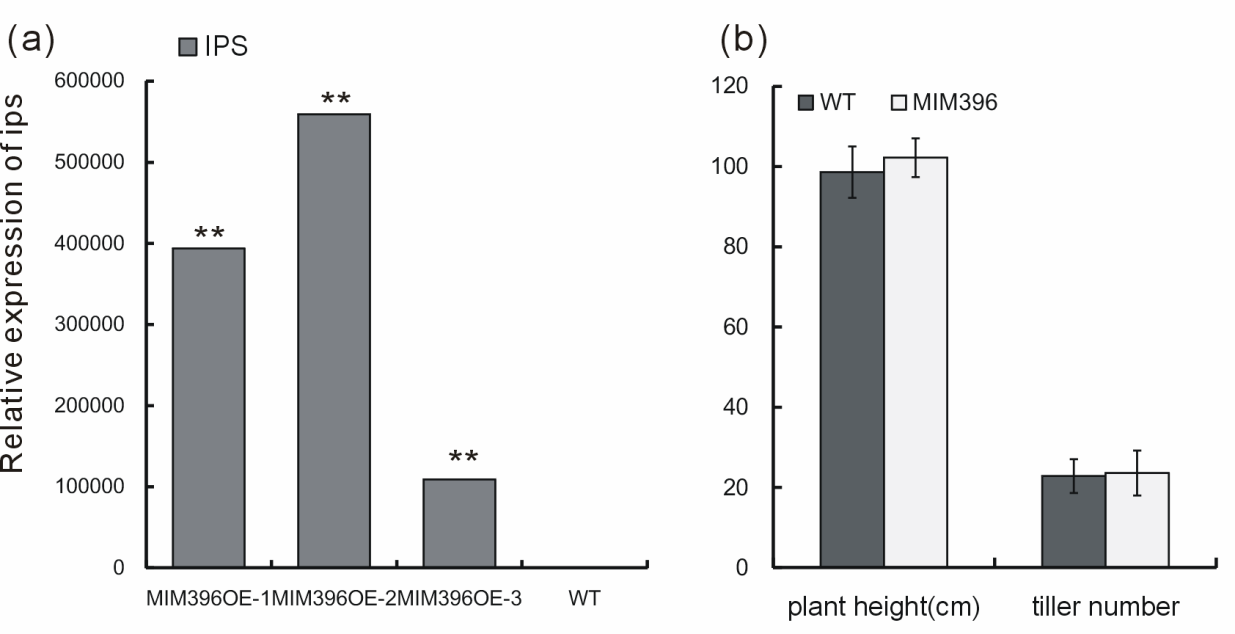


**Figure S1.** Verification and characterization of the MIM396 plants.

(a) Verification of the MIM396 plants by *ips* gene expression (n = 3). (b) Statistical analysis of plant height and tiller number of MIM396 plants compared with those of the WT (n = 20). Asterisks in (a) represent significant differences determined by Student’s *t*-test at ***P* < 0.01 compared with WT.

**
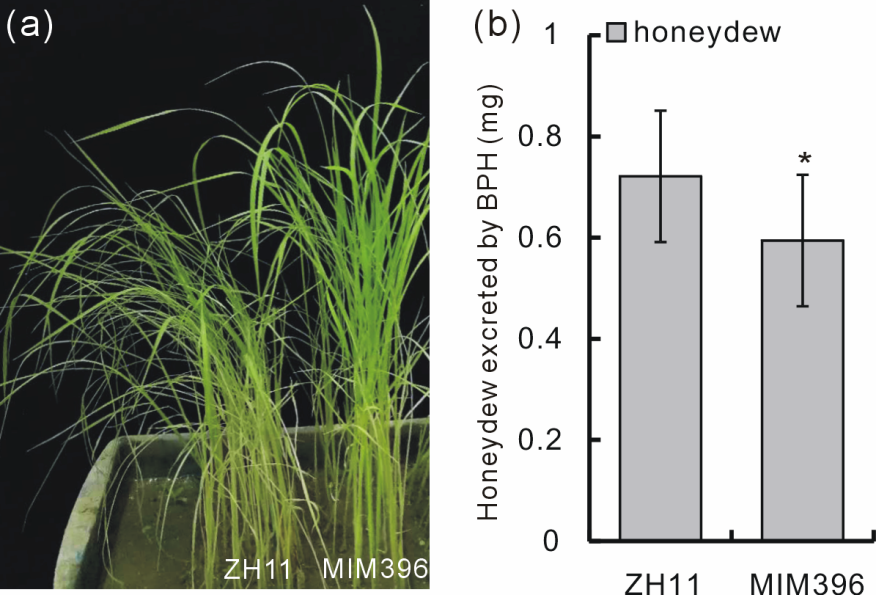
**

**Figure S2. BPH resistance of MIM396 plants in small population test (a) and in test of the contents of honeydew excreted by the BPH after feeding for 2 d (b).** Asterisk in (b) represents significant difference determined by Student’s *t*-test at **P* < 0.05.

**
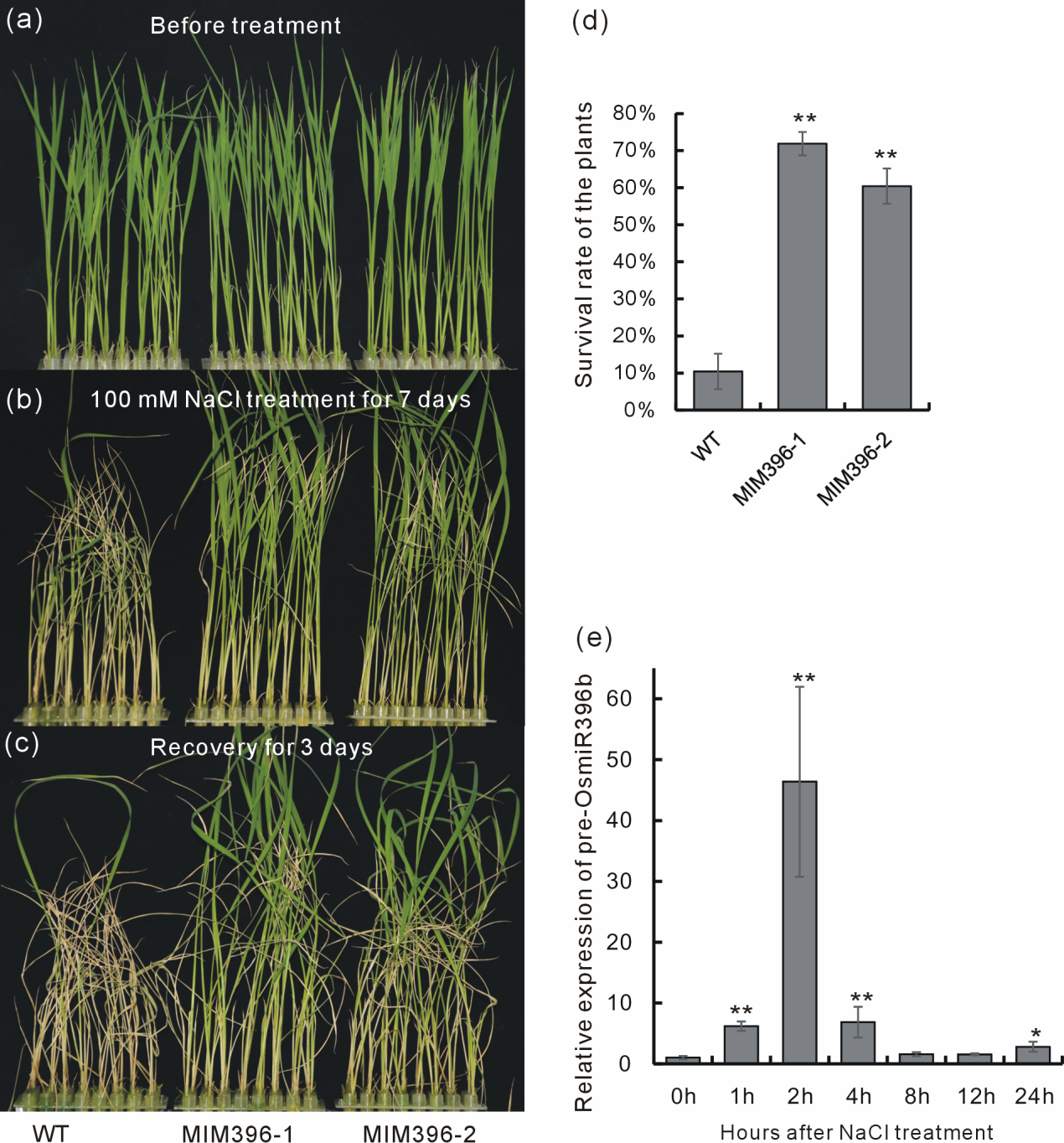
Figure S3. Tolerance of the MIM396 plants to 100 mM NaCl.**

(a) The MIM396 and WT plants cultured in 1/2 MS media before treatment of 100 mM NaCl. (b) Treatment of the MIM396 and WT plants with 100 mM NaCl for 7 d. (c) Recovery of the MIM396 and WT plants for 3 d. (d) Survival rate of the MIM396 and WT plants after NaCl treatment (n = 3). (e) qRT–PCR analysis of the transcript of OsmiR396b after 100 mM NaCl treatment (n = 3). Asterisks in (d) and (e) represent significant differences determined by Student’s *t*-test at **P* < 0.05 and ***P* < 0.01.

**
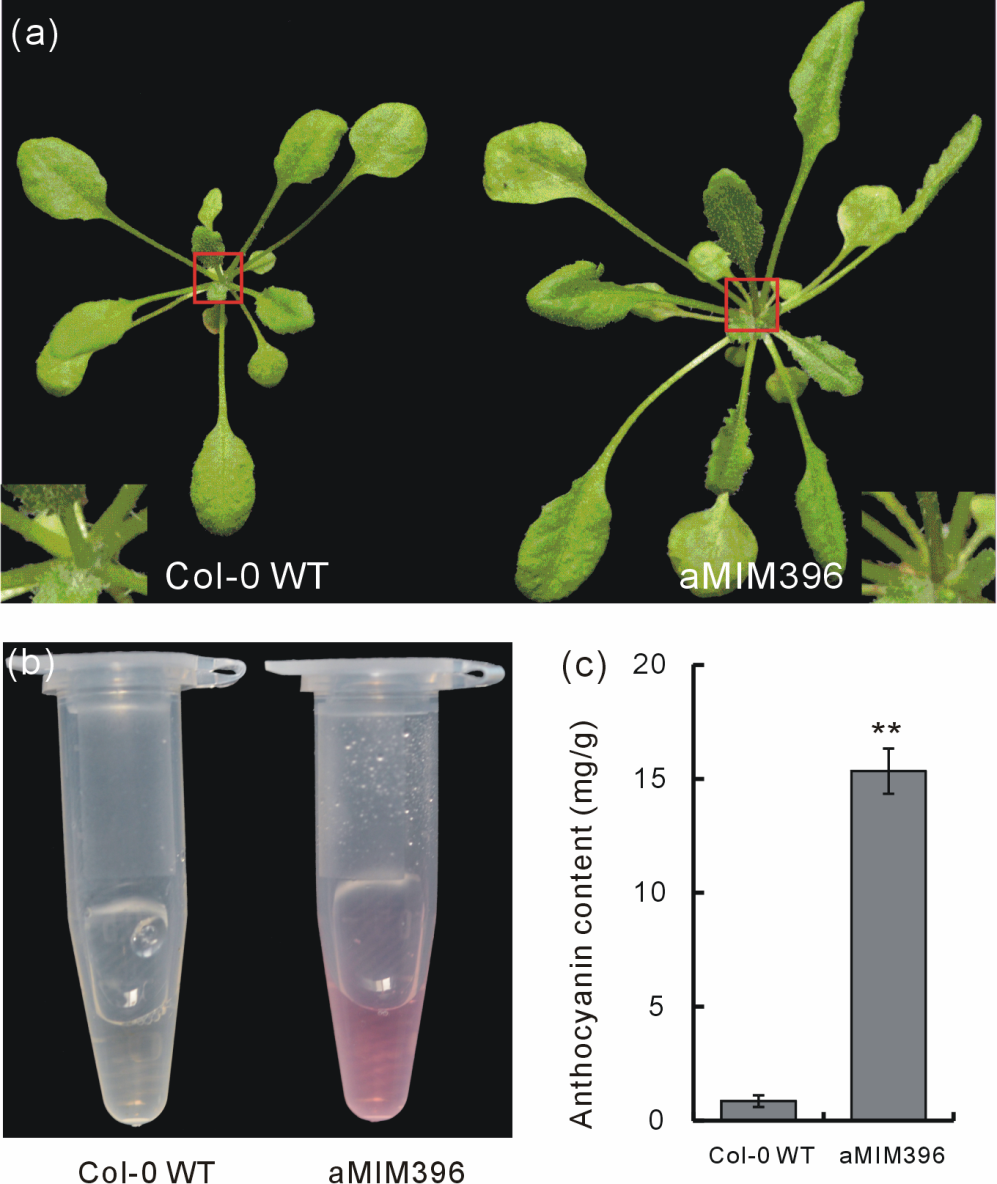
**

**Figure S4. Analysis of anthocyanin contents in the aMIM396 *Arabidopsis* plants.**

(a) The appearance of an aMIM396 plant and Col-0 WT plant, indicating the increased biomass and deep colored stems of the aMIM396 plants. Zoom-in of the respective red rectangles was inserted at the left and right bottom respectively. (b) Color of the anthocyanins extracted from the aMIM396 and Col-0 WT plants. (c) Statistical analysis of the anthocyanin contents in the aMIM396 and Col-0 WT plants (n = 3). Asterisks represent significant difference determined by Student’s *t*-test at ** *P* < 0.01.

**
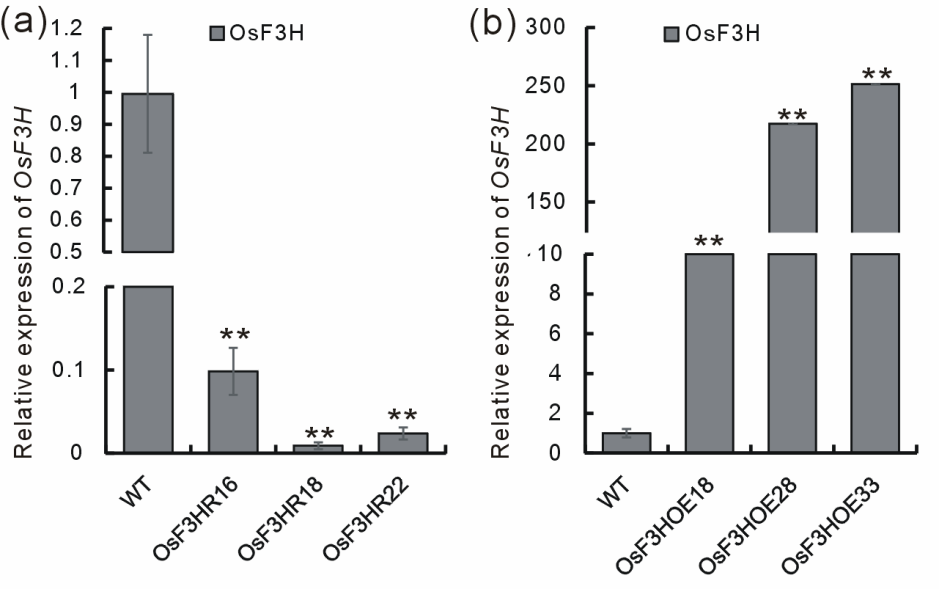
**

**Figure S5. qRT–PCR analysis of the levels of the transcript of *OsF3H* gene in OsF3HR (a) and OsF3HOE (b) plants compared with in the WT, respectively.** Asterisks represent significant differences determined by Student’s *t*-test at ***P* < 0.01 (n = 3).

**
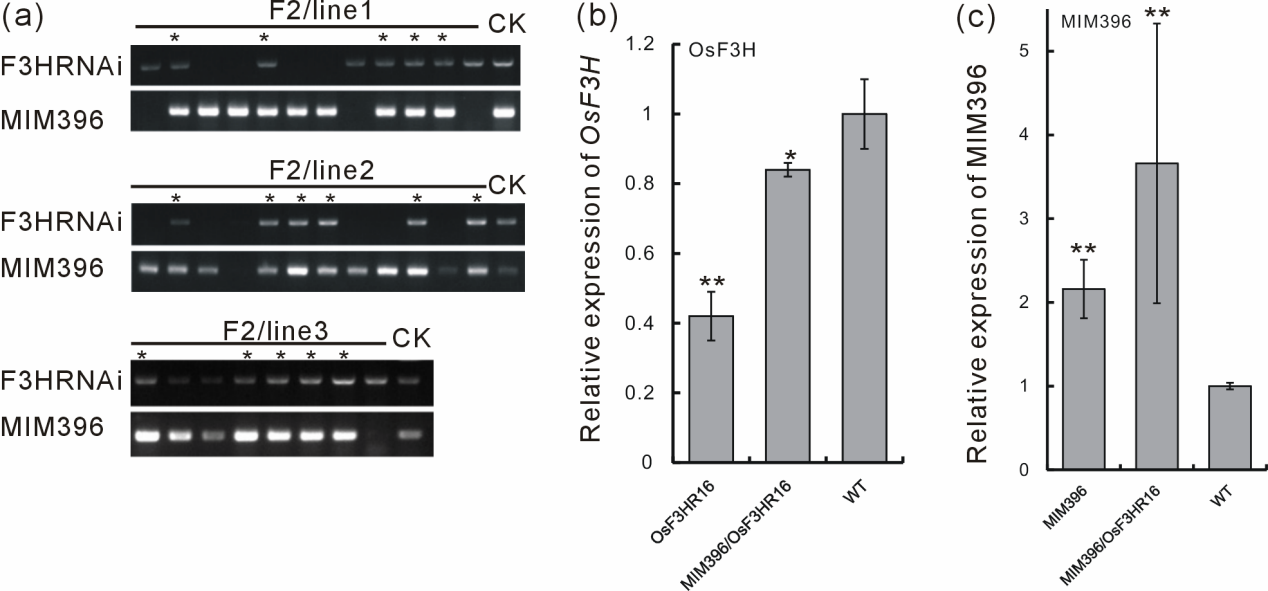
**

**Figure S6. Molecular analysis of the genetic cross between MIM396 and OsF3HR16 plants.**

(a) Genomic verification of three lines of the F2 population of the cross between MIM396 and OsF3HR16 plants. The primers used for F3HRNAi detection were F3HRNAiF and F3HRNAiR, those used for MIM396 detection were IPSF and IPSR. The stars indicated the positive hybrids. (b) qRT–PCR analysis of the transcripts of *OsF3H* in the MIM396/OsF3HR16, OsF3HR16 and WT plants (n = 3). (c) qRT–PCR analysis of the transcripts of the OsmiR396 target mimicry (MIM396) in the MIM396/OsF3HR16, MIM396 and WT plants (n = 3). Asterisks in (b) and (c) represent significant differences determined by Student’s *t*-test at ***P* < 0.01.

**
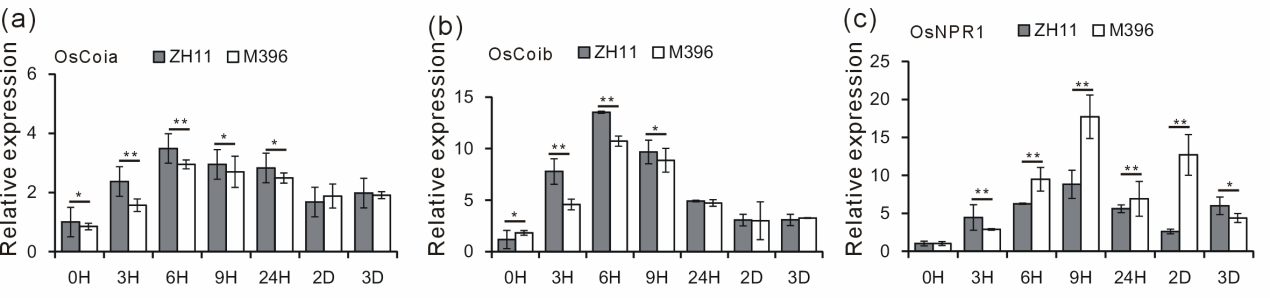
**

**Figure S7. qRT–PCR analysis of the levels of transcripts of some signaling genes in JA and SA pathway during BPH infestation in the MIM396 plants compared with those in the WT.**

(a) *OsCoia*. (b) *OsCoib.* (c) *OsNPR1*. Asterisks represent significant differences determined by Student’s *t*-test at **P* < 0.05 and ***P* < 0.01 (n = 3).
